# Rational‐Designed Principles for Electrochemical and Photoelectrochemical Upgrading of CO_2_ to Value‐Added Chemicals

**DOI:** 10.1002/advs.202105204

**Published:** 2022-01-24

**Authors:** Wenjun Zhang, Zhong Jin, Zupeng Chen

**Affiliations:** ^1^ Jiangsu Co‐Innovation Center of Efficient Processing and Utilization of Forest Resources International Innovation Center for Forest Chemicals and Materials Jiangsu Province Key Laboratory of Green Biomass‐based Fuels and Chemicals College of Chemical Engineering Nanjing Forestry University Nanjing 210037 China; ^2^ MOE Key Laboratory of Mesoscopic Chemistry MOE Key Laboratory of High Performance Polymer Materials and Technology Jiangsu Key Laboratory of Advanced Organic Materials School of Chemistry and Chemical Engineering Nanjing University Nanjing 210023 China

**Keywords:** CO_2_ conversion, electrocatalysis, photoelectrocatalysis, value‐added chemicals

## Abstract

The chemical transformation of carbon dioxide (CO_2_) has been considered as a promising strategy to utilize and further upgrade it to value‐added chemicals, aiming at alleviating global warming. In this regard, sustainable driving forces (i.e., electricity and sunlight) have been introduced to convert CO_2_ into various chemical feedstocks. Electrocatalytic CO_2_ reduction reaction (CO_2_RR) can generate carbonaceous molecules (e.g., formate, CO, hydrocarbons, and alcohols) via multiple‐electron transfer. With the assistance of extra light energy, photoelectrocatalysis effectively improve the kinetics of CO_2_ conversion, which not only decreases the overpotentials for CO_2_RR but also enhances the lifespan of photo‐induced carriers for the consecutive catalytic process. Recently, rational‐designed catalysts and advanced characterization techniques have emerged in these fields, which make CO_2_‐to‐chemicals conversion in a clean and highly‐efficient manner. Herein, this review timely and thoroughly discusses the recent advancements in the practical conversion of CO_2_ through electro‐ and photoelectrocatalytic technologies in the past 5 years. Furthermore, the recent studies of operando analysis and theoretical calculations are highlighted to gain systematic insights into CO_2_RR. Finally, the challenges and perspectives in the fields of CO_2_ (photo)electrocatalysis are outlined for their further development.

## Graphical abstract

CO_2_‐to‐fuel conversion is considered as a promising strategy for decreasing CO_2_ concentration and further upgrading to chemical feedstocks. Accordingly, this review compares the catalytic technologies and summarizes the recent development of advanced catalysts for highly‐efficient CO_2_ conversion through electrocatalysis and photothermocatalysis during the past 5 years. In addition, the catalytic mechanisms, challenges, and perspectives of these technologies are emphasized.

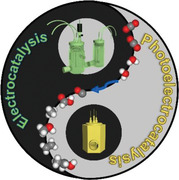

## 1 Introduction

**Figure 1:**
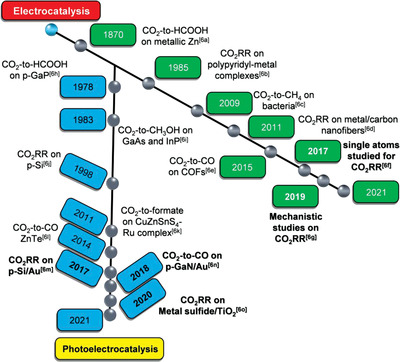
Chronology of the electro‐ and photoelectrochemical CO_2_ conversion. The research progresses in the recent 5 years (2016–2021) are highlighted in bold.^[^ ^6^ ^]^

The increasing CO_2_ emission in the ambient air has caused deteriorative global warming and ocean acidification problems. Accordingly, CO_2_ conversion has been regarded as an effective approach to alleviate atomospheric CO_2_ concentration in the past decades, aimed at maintaining the ecosystem carbon cycle.^[^ ^1^ ^]^ Inspired by natural photosynthesis, CO_2_ capture and utilization via chemical reaction path have become a hot research topic in recent years, especially advanced technologies and rational‐designed catalysts have attracted wide attention to maximize the efficiency of CO_2_ conversion.^[^ ^2^ ^]^ Traditional thermocatalytic CO_2_ transformation as a popular approach has been developed to reduce CO_2_ and further upgrade to important chemicals and fuels in an industrial scale. However, the application of thermocatalytic process is significantly affected by the excess combustion of fossil fuels, which can severely interrupt the carbon cycle and sustainable energy production.^[^ ^3^ ^]^ Hence, as clean and renewable energy techniques, electro‐ and photoelectrocatalysis have emerged for CO_2_ reduction reaction (CO_2_RR), which can be processed in a mild reaction condition (i.e., room temperature and ambient pressure) to realize CO_2_ reduction to value‐added chemicals production.^[^ ^4^ ^]^ Notably, CO_2_ as an inert molecule requires excessive energy input to activate CO_2_, and thus highly‐efficient catalysts are required to provide active sites for the adsorption and transformation of CO_2_ molecules.^[^ ^5^ ^]^ The chronology of the electro‐ and photoelectrocatalytic CO_2_ conversion is shown in **Figure** **1**, emphasizing the milestones of the developments of remarkable catalysts.^[^ ^6^ ^]^

Electrocatalytic CO_2_RR makes use of aqueous electrolytes as the hydrogen source rather than the molecular H_2_ in thermocatalysis. When applied with different equilibrium potentials, CO_2_ molecules can be converted into small carbonaceous molecules via a multiple‐electron transfer mechanism, such as, formate, CO, alkanes, alcohols, and other hydrocarbons. The different classes of products for CO_2_ electroreduction attribute to the various formation energy barriers and adsorption abilities of different intermediates, which have also been described in our previous review.^[^ ^7^ ^]^ Briefly, formate can be easily obtained from *OCHO formation and *HCOOH desorption while CO is generated from *COOH formation and *CO desorption. Notably, *CO is also an important intermediate in CO_2_RR to higher‐order hydrocarbons, aldehydes, and alcohols via *CO dimerization and subsequent hydrogenation process.^[^ ^8^ ^]^ However, C = O activation, the competing hydrogen evolution reaction (HER), and the separation of mixed products have remained inevitable challenges during CO_2_RR. Accordingly, constructing highly active interfaces and/or engineering the electronic/geometric properties of catalysts have been widely employed to realize highly active and selective CO_2_ hydrogenation and reduction.^[^ ^9^ ^]^

**Figure 2:**
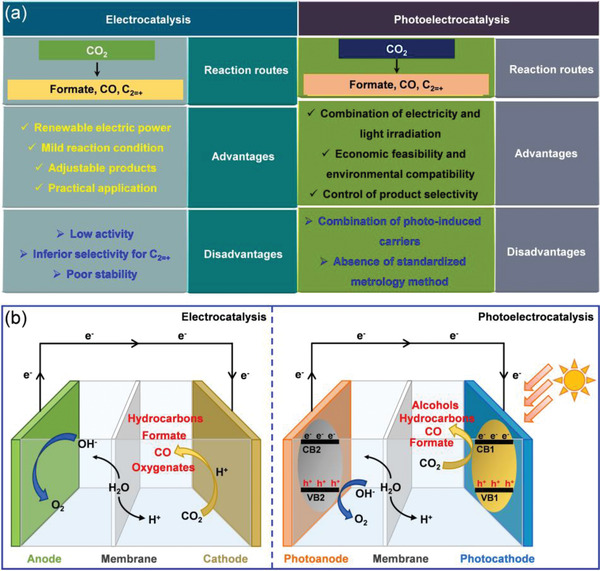
a) Characteristics of the technologies for electro‐ and photoelectrocatalytic CO_2_ conversion. b) Schematic illustration of the working principles of the full‐cell type (photo‐)electrochemical CO_2_ conversion systems. Reproduced with permission.^[^ ^14^ ^]^ Copyright 2020, American Chemical Society.

Renewable solar energy as an ideal alternative can provide extra energy for electrocatalytic processes. Therefore, the integration of light and electricity in the CO_2_ catalysis emerges as an intelligent approach to promote the conversion efficiency of CO_2_ and further reduce the consumption of fossil fuels.^[^ ^10^ ^]^ Semiconductors regarded as a type of promising material realize the combination of solar and electronic energy, which can be applied not only as catalysts but also as light harvesters in photoelectrocatalysis.^[^ ^11^ ^]^ Under the sunlight irradiation, semiconductors can accelerate the C = O activation process, which reduces CO_2_ to hydrocarbons by the photogenerated carriers.^[^ ^12^ ^]^ Specifically, a semiconductor photocatalyst with a suitable band structure is of great significance to break the C = O bond. The bottom of the conduction band of a semiconductor should be more negative than the CO_2_ reduction potential, while the top of the valence band should be more positive than the water oxidation potential, which could thereby simultaneously realize CO_2_‐to‐fuels conversion and oxygen evolution reaction (OER). Notably, photoelectrocatalysis extends the material choices in comparison to photocatalysis, which results in a higher potential to meet the requirements of suitable band position and redox potential level for CO_2_ conversion with the assistant of an external bias. Meanwhile, photoelectrocatalysis provides extra light energy to decrease the overpotentials for electrocatalytic CO_2_RR, which can also enhance the lifespan of photo‐induced carriers in the whole catalytic process. Accordingly, some approaches (e.g., structure engineering, cocatalyst doping, and heterojunction design) have been employed to optimize the activity of catalysts, and thereby generate different carbonaceous compounds like CO, formate, alcohols, and hydrocarbons.^[^ ^13^ ^]^ In **Figure** **2**, we summarize the major characteristics and working principle of the full‐cell type of electro‐ and photoelectrocatalysis toward CO_2_ conversion.^[^ ^11^, ^12^, ^14^ ^]^

Herein, we present a timely and comprehensive review of the recent advances in electro‐ and photoelectrocatalytic CO_2_ conversion during the past 5 years. First, the motivation and fundamentals of the catalytic technologies are mentioned. Then, since the developments of catalysts play a significant role in CO_2_ electro‐ and photoelectrocatalysis, the important advances in design principles of catalysts have been emphasized in this review. There are some key points mentioned on synthetic strategies for preparing the (photo)electrodes and the main factors for improving the structure‐activity relationships in these catalytic systems. Thirdly, the deep understanding of catalytic mechanism and reaction pathways of CO_2_ reduction are discussed, mainly based on advanced techniques of in situ/operando characterizations and theoretical calculations. Last but not least, the challenges and future perspectives are addressed to promote the highly‐efficient CO_2_ utilization and upgradation, aimed at realizing the feasibility for industrial production. We hope this review can shed a light on the chemical transformation of CO_2_ molecules with the help of clean and renewable energy, and provide sufficient inspiration for researchers in this exciting field.

## 2 Electrocatalysis

### 2.1 Motivation and Principles

**Figure 3:**
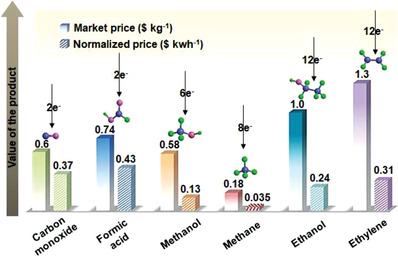
Comparisons of the market prices ($ kg^–1^) and added values per kWh electrical energy input ($ kWh^–1^) values of the representative hydrocarbons. Reproduced with permission.^[^ ^15^ ^]^ Copyright 2019, Wiley‐VCH.

**Figure 4:**
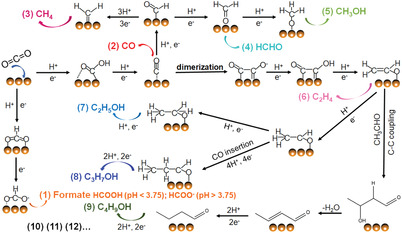
Possible reaction pathways for the generation of formate, CO, and other hydrocarbons in ECR.

Although CO_2_ thermocatalysis is the most popular catalytic route for large‐scale industrial manufacture, whereas the enormous energy demand is often supplied via the excessive calcination of non‐renewable fossil fuels. Therefore, converting CO_2_ into fuels and chemicals in a more sustainable manner that is driven by renewable energy technologies, has gained extensive attention. Since the cost of electricity generated from solar, wind, and other clean energy sources continuously declines, electrocatalysis emerges as a greener strategy for CO_2_ reduction and gains enormous attention in recent years. CO_2_ is a thermodynamic stable molecule that requires excess energy of 806 kJ mol^−1^ to activate the C = O bond. Moreover, the first proton‐coupled electron transfer process needs to overcome high energy barriers to form the surface‐adsorbed species (i.e., *COOH and *OCHO intermediates). Accordingly, the external potentials should be applied to realize CO_2_ activation and conversion at reasonable rates. However, different types of carbonaceous compounds could be simultaneously generated through multi‐electrons (2, 4, 6, 8, 12, or even more) transfer pathways in CO_2_ reduction due to the close thermodynamic redox potentials of these possible products (**Table** **1**). As observed from **Figure** **3**, formic acid and CO are the higher value‐added products in terms of electrical energy input, when compared with other hydrocarbons (e.g., MeOH, CH_4_, ethanol, and ethylene).^[^ ^15^ ^]^ On the other hand, the product selectivities would decrease dramatically when generating the molecules that require more electrons. Thus the economic benefits of electrochemical CO_2_ reduction (ECR) to different products depend on not only the market demand of the higher‐value‐added chemicals (C_2_‐C_4_) but also the product separation costs, the further decrease of electricity costs, and the improvement of the electrocatalytic activity. Accordingly, different reaction pathways toward ECR were studied experimentally and theoretically in virtue of the applied electrocatalysts and reaction conditions, resulting in significantly different product distributions, as illustrated in **Figure** **4**.^[^ ^7^, ^16^ ^]^ First, CO_2_ molecules are adsorbed on the surface of catalysts and then activated via the proton‐coupled electron transfer process to generate active intermediates (i.e., *OCHO and *COOH). Specifically, *OCHO species will form once the O atom of the activated *CO_2_ species binds to the electrocatalyst surface, and meanwhile, the C atom is protonated. Another case is that the C atom of *CO_2_ binds to the surface of the electrocatalyst, and the O atom is protonated to obtain *COOH intermediate. Once the electron‐proton pairs are transferred, three pathways can involve: the first pathway is that the formic acid (pH < 3.75) or formate (pH > 3.75) generates at different pH values; whereas the second pathway results in CO formation after the *CO intermediate desorbed from the surface of the catalyst. Meanwhile, Cu‐based catalyst dominates the third pathway toward CO_2_‐to‐hydrocarbons/alcohols conversion. When *CO binds tightly to the catalyst surface, dimerization would happen to form the *C(O)(O)C* species at low overpotentials. On the other hand, *CO will be hydrogenated into *CHO intermediates at relatively high overpotentials, and therefore generating CH_4_, HCHO, CH_3_OH, C_2_H_4_, and other C_2_/C_2+_ chemicals. The selectivities of different hydrocarbon production are closely related to the binding strength of *OCHO, *COOH, and *CO intermediates. Meanwhile, the free protons or proton donators escaped from aqueous electrolyte possibly convert to *H species, which is regarded as the immediate toward undesirable H_2_ by‐product at the electrode surface. Herein, high‐performance electrocatalysts should be carefully engineered to obtain ideal carbonaceous products with satisfactory selectivity in ECR.

**Table 1:** The standard potentials (*E* ^0^) of possible half‐reactions of electrochemical CO_2_ reduction in aqueous solutions for the different hydrocarbon products at 25 °C, 1 atm, and pH 7; Reproduced with permission.^[^ ^15^ ^]^ Copyright 2019, Wiley‐VCH

Possible half‐reactions of ECR	*E* ^0^ (V vs SHE)
CO_2_ (g) + e^–^ → CO_2_^•–^	−1.90
CO_2_ (g) + 2H^+^ + 2e^–^ → HCOOH (l)	−0.55
CO_2_ (g) + 2H^+^ + 2e^–^ → CO (g) + H_2_O (l)	−0.52
CO_2_ (g) + 4H^+^ + 2e^–^ → HCHO (l) + H_2_O (l)	−0.48
CO_2_ (g) + 6H^+^ (l) + 6e^–^ → CH_3_OH (l) + H_2_O (l)	−0.38
CO_2_ (g) + 8H^+^ + 8e^–^ → CH_4_ (g) + 2H_2_O (l)	−0.24
2CO_2_ (g) + 12H^+^ + 12e^–^ → C_2_H_4_ (g) + 4H_2_O (l)	−0.38
2CO_2_ (g) + 12H^+^ + 12e^–^ → C_2_H_5_OH (l) + 3H_2_O (l)	−0.35
2CO_2_ (g) + 14H^+^ + 14e^–^ → C_2_H_6_ (l) + 4H_2_O (l)	−0.28
3CO_2_ (g) + 18H^+^ + 18e^–^ → C_3_H_7_OH (l) + 5H_2_O (l)	−0.30

### 2.2 Plausible Routes for CO_2_ Conversion

Depending on the ECR products, the applied catalysts can be categorized into three classes to realize CO_2_ activation and conversion. The following contents mainly focus on these pathways, and the special attention lies on the developed heterogeneous catalysts (e.g., metals, carbon materials) since the homogeneous ones always realize ECR in aprotic solution (for example, expensive ionic liquid) and are unsuitable for practical applications.

#### 2.2.1 CO_2_ to Formate/Formic Acid

**Figure 5:**
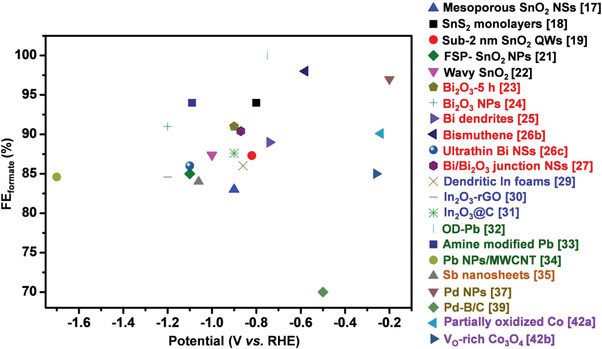
Comparison of the optimal FE_formate_ values toward different nanostructured catalysts (e.g., Sn, Bi, In, Pb, Sb, Pd, Co) in the recently published literature.

The conversion of CO_2_ to formate/formic acid has been regarded as an ideal choice for hydrogen carriers and HCOOH fuel cells, due to the advantages of easy storage and high safety. Realizing CO_2_ electroreduction into formate/formic acid involves two elementary steps. First, CO_2_ molecules go through a proton‐electron pair transfer process to obtain an *OCHO intermediate. Then, the *OCHO intermediate further converts to *HCOOH via the subsequent proton‐electron transfer at pH < 3.75 and thus generating formate/formic acid that can easily escape from the catalyst surface. However, the products could exist in the deprotonated form (HCOO^−^) at pH > 3.75. In recent five years, nanostructured metals like Sn, Bi, In, Pb, Sb, Pd, Co have been reported to be promising candidates for formate/formic acid production in ECR (**Figure** **5**). The special focuses lie in the studies of their nanostructures, index planes, vacancies/defects, grain boundaries, and supports to achieve a high activity, satisfying selectivity, and long‐term stability.

**Figure 6:**
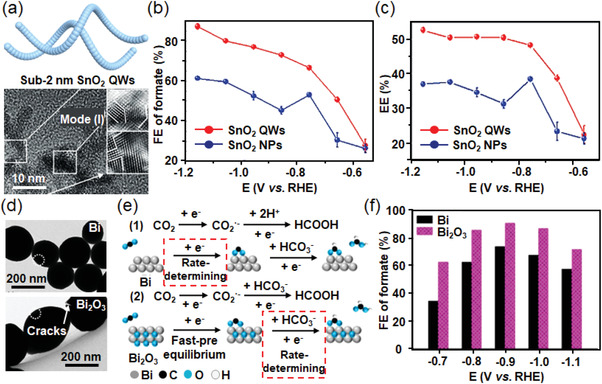
a) Structural illustration and HRTEM image of sub‐2 nm ultrathin SnO_2_ quantum wires. b) FE_formate_ and c) EE of ultrathin SnO_2_ quantum wires and SnO_2_ NPs. Reproduced with permission.^[^ ^19^ ^]^ Copyright 2019, Wiley‐VCH. d) TEM and HRTEM images of Bi_2_O_3_ catalyst. e) Proposed pathways of CO_2_ reduction to formate on Bi and Bi_2_O_3_ catalysts. f) FE_formate_ of Bi and Bi_2_O_3_ catalyst as the function of potentials in ECR. Reproduced with permission.^[^ ^23^ ^]^ Copyright 2019, American Chemical Society.

**Figure 7:**
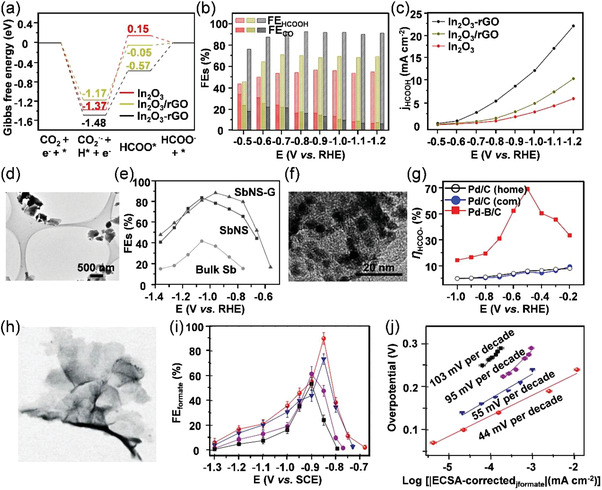
a) Gibbs free energy diagrams for CO_2_ reduction to formate on In_2_O_3_‐rGO hybrid, In_2_O_3_/rGO, and In_2_O_3_ catalysts. b) FE_CO_, FE_formate_, and c) *J*_formate_ for In_2_O_3_–rGO hybrid, In_2_O_3_/rGO, and In_2_O_3_ catalysts. The color codes in (c) apply to (b). Reproduced with permission.^[^ ^30^ ^]^ Copyright 2019, American Chemical Society. d) TEM image of Sb nanosheets. e) FE_formate_ values of bulk Sb, Sb nanosheets, and Sb nanosheets‐graphene composite catalysts at different potentials. Reproduced with permission.^[^ ^35^ ^]^ Copyright 2017, Wiley‐VCH. f) TEM image and the size distribution histogram of Pd‐B/C catalyst. g) Potential dependent FE_formate_ of Pd‐B/C, Pd/C (home), and Pd/C (com) catalysts. Reproduced with permission.^[^ ^40^ ^]^ Copyright 2019, John Wiley & Sons, Inc. h) TEM image of partially oxidized Co 4‐atom‐thick layers. i) FE_formate_ at each applied potential and j) ECSA‐corrected Tafel plots for formate production of partially oxidized Co 4‐atom‐thick layers (red), Co 4‐atom‐thick layers (blue), partially oxidized bulk Co (violet), and bulk Co (black). Reproduced with permission.^[^ ^42a^ ^]^ Copyright 2016, Springer Nature Limited.

I) Sn‐based materials have attracted wide attention owing to their abundance and environment‐friendly properties. Many studies have concentrated on the optimal design of metallic Sn, Sn oxides, and Sn sulfides experimentally and theoretically. For example, mesoporous SnO_2_ nanosheets were fabricated by a self‐templated synthetic method,^[^ ^17^ ^]^ which possesses a large surface area and 3D hierarchical nanostructure, resulting in numerous undercoordinated sites or structural defects. Owing to the above merits, mesoporous SnO_2_ nanosheets exhibited small overpotential (710 mV), high faradaic efficiency of formate (FE_formate_ = 83.0%), and long‐term stability in ECR. SnS_2_ monolayers were synthesized by He et al. via Li‐intercalation and exfoliation process, which exhibited an extraordinary FE_formate_ up to 94% and excellent durability over 80 h in ECR.^[^ ^18^ ^]^ The atomic‐scale thickness accelerated the proton‐electron transfer efficiency and the formation of *OCHO and *HCOOH intermediates, which were subsequently transformed into formate via a two‐electron transfer pathway. In addition, the introduction of grain boundary could break the local spatial symmetry of materials, which significantly optimizes the binding energies of reaction intermediates, and thus realizes a high selectivity and energy efficiency for CO_2_‐to‐formate conversion. Inspired by this, sub‐2 nm ultrathin 1D SnO_2_ quantum wires constructed by numerous quantum dots were fabricated,^[^ ^19^ ^]^ presenting abundant grain boundaries on the catalyst surface (**Figure** **6**a). The as‐prepared SnO_2_ quantum wires exhibited a peak value of FE_formate_ (87.3%) and energy efficiency (EE = 52.7%). Moreover, the FE_formate_ and EE_formate_ could maintain above 80% and 50% in a wide potential window, respectively (Figure 6b,c). Furthermore, the creation of structural defects such as oxygen vacancies in the catalysts was found to effectively improve CO_2_ activation, electron mobility, the interaction between reaction intermediates and active sites, as well as desorption ability of *HCOOH intermediates.^[^ ^20^ ^]^ Daiyan et al. employ the industrially adopted flame spray pyrolysis technique to synthesize SnO_2_ nanoparticles with the active oxygen hole center,^[^ ^21^ ^]^ realizing a high FE_formate_ of 85% and a current density of −23.7 mA cm^−2^ at a potential of −1.1 V versus RHE. Similarly, Chen's group reported an “all in one” wavy SnO_2_ network catalyst,^[^ ^22^ ^]^ which simultaneously possessed grain boundaries, oxygen vacancies, and low‐coordinated active edge/corner sites. With the optimization of surface structures, wavy SnO_2_ catalyst showed maximum FE_formate_ of 87.4% and EE_formate_ of 57.5% at −1.0 V versus RHE.
II) The potential of metallic Bi has recently been explored for highly selective formate production from ECR in an aqueous solution, which used to be commonly applied to CO_2_‐to‐CO conversion in aprotic electrolytes before 2016. Element Bi locates close to the Sn in the periodic table and possibly provides similar electronic properties in ECR. Moreover, metallic Bi could suppress the competitive H_2_ evolution since it exhibits high free energy of hydrogen adsorption (Δ*G*_H_). Bi‐based catalysts always contain oxygen species, which are inevitably introduced during the synthetic process. Accordingly, Deng et al. reported a Bi oxides catalyst with a high concentration of Bi‐O species by applying a time‐dependent oxidation treatment.^[^ ^23^ ^]^ The roles of the Bi‐O species toward highly selective formate production in ECR were analyzed experimentally and theoretically (Figure 6d). The Bi‐O structure was found to facilitate CO_2_ adsorption and accelerate the rate‐determining step (namely, *CO_2_^•−^ + *H → *OCHO) by lowing the free energy for intermediate formation (Figure 6e). Therefore, the resulting Bi_2_O_3_ catalyst showed a maximum FE_formate_ of 91% and a partial current density of ≈8 mA cm^−2^ at −0.9 V versus RHE (Figure 6f). To further improve the durability of metastable Bi oxides, the impact of the morphology of Bi_2_O_3_ was investigated, demonstrating that the optimized Bi_2_O_3_ nanoparticles could achieve improved FE_formate_ of 91% at the applied potential of −1.2 versus RHE and stable performance over 23 hours.^[^ ^24^ ^]^ To design a high‐performance Bi‐based catalyst for ECR, the coordinately unsaturated sites such as high‐indexed planes and edge/corner sites can be incorporated onto the surface of the Bi electrodes, which enhances the stabilization of reaction intermediates onto the catalyst surface, and thus promotes CO_2_ conversion. Inspired by this, some nanostructured Bi catalysts with different morphology have been explored, for example, nanodendrites, nanosheets, and monolayers to completely expose low‐coordinated sites for CO_2_ reduction. Experimental results demonstrated that Bi dendrites realized a high selectivity for formate formation (FE_formate_ = ≈89%) and long‐term durability (12 h) at a moderate potential (−0.74 V vs RHE).^[^ ^25^ ^]^ Theoretical calculations were further conducted to verify that the high‐indexed planes exposed on the Bi dendrites preferred the formation and stabilization of *OCHO species, which were important reaction intermediates for CO_2_‐to‐formate conversion. In addition, bulk Bi stacks in a similar way as black phosphorus, which can be easily exfoliated into monolayers or few layers with abundant active edge sites and large surface areas. Accordingly, some studies have been recently sprung up to synthesize 2D Bi nanosheets derived from the in/ex situ topotactic transformations of Bi oxyhalide, liquid‐phase exfoliation of commercial bulk Bi and wet chemical synthesis of Bi chloride.^[^ ^26^ ^]^ These ultrathin Bi nanosheets with monolayer or few layers achieved highly efficient ECR performance toward formate production, accompanied with promising activity and stability at a moderate potential, and even satisfactory selectivity over a broad potential window. In addition to the design of unique nanostructures, endowing catalysts with effective componential features can also optimize their catalytic performance in ECR. Wu and co‐workers synthesized the defect‐rich Bi/Bi_2_O_3_ nanosheets directly on the carbon fiber papers.^[^ ^27^ ^]^ The construction of Bi/Bi_2_O_3_ junction interfaces could bring extra electronic effects to enhance the processes of CO_2_ activation and electro‐proton transfer, as well as the stabilization of reduction intermediates, which contributes to a maximum FE_formate_ value of 90.4% at −0.87 V versus RHE. Combining Bi‐based nanostructures with highly conductive carbon supports has also been regarded as an effective method to optimize the activity of ECR. Barik's group found that highly‐dispersed BiOCl species on N‐doped carbon composites synergistically performed a maximum FE_formate_ of 84.3% at −0.87 V versus RHE in an aqueous solution, which possibly resulted from the rapid electron transfer, increased CO_2_ adsorption, and short diffusion pathway of reactant.
III) Metallic In has a relatively higher cost than that of Sn and Bi, therefore attracting limited attention for large‐scale applications. However, as an environmental‐friendly metal, indium is one of the earliest investigated main group metals for formate formation toward ECR in a similar way as Sn and Bi. Bulk In reached a FE_formate_ value of ≈95% at −1.55 V versus RHE in the 1990s,^[^ ^28^ ^]^ unfortunately the required overpotential (1.36 V) and current density (5 mA cm^−2^) were unsatisfactory. To optimize the activity of In‐based catalysts, dendritic In foams have been recently prepared by an electrodeposition method in an aqueous solution with the existence of Cl^−^ ions when using the hydrogen bubble dynamic templates.^[^ ^29^ ^]^ The as‐obtained In electrode possesses a large electrochemical surface area (ECSA) and needle‐like dendrite nanostructures, showing an improved FE_formate_ value of 86% at a relatively low potential of −0.86 V versus RHE. Since an oxide layer is usually inevitable on the surface of In under ambient conditions, In_2_O_3_ has also been regarded as the active species that benefit the CO_2_ adsorption and intermediate binding capacity in ECR. Meanwhile, the close interactions between different components in a hybrid material offer synergistic effects for enhanced CO_2_ electroreduction activity. Based on these proposed strategies, Zeng's group synthesized a hybrid catalyst that is composed of porous In_2_O_3_ nanobelts and reduced graphene oxide (In_2_O_3_‐rGO) by a facile two‐step process.^[^ ^30^ ^]^ The theoretical calculations and electrochemical microkinetic analysis demonstrated that the presence of chemical coupling within the In_2_O_3_‐rGO hybrid caused the changes in electronic density, contributing to the higher extent of electron transfer on the catalyst surface. Accordingly, the electron‐rich structure of In_2_O_3_‐rGO significantly affected the adsorption ability of reaction intermediates, therefore facilitating the formation of key intermediate *HCOO^−^ during CO_2_‐to‐formate conversion. The aforementioned results clarified the experimental observations that In_2_O_3_‐rGO hybrid exhibited enhanced FE_formate_ (1.4‐fold) and current density (3.6‐fold) than that of bare In_2_O_3_ nanobelts (**Figure** **7**a–c). Similarly, Mou and co‐workers reported an In_2_O_3_@C catalyst,^[^ ^31^ ^]^ which showed the peak FE_formate_ value of 87.6% and satisfactory durability (12 h) at an overpotential of only 710 mV. These performances were significantly superior to that of In_2_O_3_ nanoclusters due to the increased ECSA and the positive effects of carbon black.
IV) Pb‐based catalysts perform promising activity and selectivity for formate formation in ECR, whereas its high toxicity brings adverse impacts on human health and the environment, and thus hindering their practical applications. Kanan's group prepared PbO_2_‐derived nanocrystalline Pb films by in situ electrochemical reductions,^[^ ^32^ ^]^ which significantly suppressed HER and reached FE_formate_ values above 90% from −0.7 to −1.0 V versus RHE. Importantly, the metastable Pb oxide (hydroxide) species covered on Pb surface favor the ECR process and act as passivation layers for HER. To simultaneously realize a higher current density with an optimal FE_formate_, arylaliphatic amines, aminophenyl, and nitrophenyl derived Pb catalysts were synthesized.^[^ ^33^ ^]^ The enhanced CO_2_‐to‐formate performance was due to the effectively improved CO_2_ capture, suppressed H_2_ generation, and lowered overpotential. Accordingly, the amine‐derived Pb electrocatalysts showed FE_formate_ of 94% and a partial current density for formate production (*j*_formate_) of 9.5 mA⋅cm^−2^ at an applied potential of −1.09 V versus RHE. To further fabricate an efficient catalyst with enhanced electrical conductivity, and larger specific surface area, a multi‐walled carbon nanotube aerogel supported Pb catalyst was synthesized,^[^ ^34^ ^]^ which could provide a rapid mass transfer pathway for reactants and lead to sufficient contact between CO_2_ molecules and active sites. Finally, the as‐obtained electrocatalyst performed high FE_formate_ (84.6%) and large current density (28 mA cm^−2^) with long‐term stability (10 h).
V) Sb is closed to metallic Sn and Bi in the periodic table, while it remains unexploited for ECR. This is probably due to the limited active sites on the surface of bulk Sb, which have a strong impact on its practical application in the catalytic field. Interestingly, the pristine Sb material possesses a rhombohedral layered structure, which can be easily exfoliated into 2D form (namely, antimonene). Due to its satisfactory stability and specific features, researchers have preliminarily tried to study its physiochemical properties and explored the catalytic performance. Li and co‐workers successfully synthesized 2D Sb nanosheets with few layers by cathodic exfoliation,^[^ ^35^ ^]^ which exposed a higher density of active edge sites for CO_2_ reduction (Figure 7d). The Sb nanosheets realized a peak value of FE_formate_ (≈84%) at a moderate overpotential of 0.97 V. To further enhance FE_formate_ and current density of Sb nanosheets, anodically exfoliated graphene nanosheets were added to form an Sb nanosheets‐graphene composite, which exhibited a higher FE_formate_ value of 88.5% and a *j*_formate_ above 8 mA cm^−2^ at a decreased overpotential of 0.87 V for a long‐term testing (>12 h). The improved CO_2_‐to‐formate behavior mainly resulted from the abundant active sites on 2D Sb nanosheets and strong interactions between Sb and graphene (Figure 7e).
VI) Pd‐based catalysts have also been applied in electrocatalytic fields, performing satisfactory selectivity of C_1_ compounds production in ECR.^[^ ^36^ ^]^ Detailed mechanisms have been studied to analyze the keys that dominate the activity and selectivity of catalysts. The optimized morphology and composition of nanostructured Pd catalysts, as well as, the in situ formed active phases (e.g., Pd‐hydride) caused by applied potentials resulted in various selectivities toward reduction products via distinct reaction pathways and intermediates. Moreover, the introduction of heteroatoms (e.g., N, B) and supports (e.g., metal oxides, carbons) can improve the activity and selectivity of Pd‐based catalysts and further maintain good durability for long‐term ECR. Sargent's group prepared different morphologies of Pd nanoparticles to expose high‐density high‐index surfaces,^[^ ^37^ ^]^ which optimized the binding capability of intermediates and thus realized preferable activity and selectivity of CO_2_‐to‐liquid fuels (mainly, formate) conversion. The as‐obtained Pd nanoparticles exhibited a high FE_formate_ value of 97% with a record current density of 22 mA cm^−2^ at a relatively low overpotential of −0.2 V. The abundant higher‐index facets exposed on the Pd surface provided more step sites and undercoordinated atoms, which effectively decreased the energy barrier of reaction intermediates (HCOO*) formation favoring formate production. By altering active phases of Pd nanoparticles under different applied potentials with the existence of *H species, Gao et al. found that different products would be generated via a specific reaction pathway and preferable intermediates through advanced analysis of in situ X‐ray absorption spectroscopy, in situ attenuated total reflection‐infrared spectroscopy, and DFT results.^[^ ^38^ ^]^ The Pd catalyst contains a core–shell structure (*α*+*β* PdH*_x_* @PdH*_x_*) above −0.2 V versus RHE, which mainly generate formate via the HCOO* intermediate, whereas the in situ formed *β‐*PdH*_x_* @Pd below −0.5 V versus RHE prefers CO production via the COOH* intermediate. Jiang and co‐workers reported a boron‐doped Pd catalyst (Pd‐B/C),^[^ ^39^ ^]^ which effectively suppressed the CO poisoning phenomenon and selectively facilitated CO_2_‐to‐formate conversion. During ECR, Pd‐B alloy would form once B atoms are inserted in the Pd lattice, and favors the formation of HCOO* intermediates. Accordingly, the FE_formate_ value achieves ≈70% over 2 h electrolysis at a low voltage of −0.5 V versus RHE on the Pd‐B/C catalyst (Figure 7f,g). To broaden the potential window of Pd catalyst toward ECR, N, B‐codoped TiO_2_ nanotubes were fabricated and employed as a support to optimize the activity and selectivity of metallic Pd in CO_2_ reduction.^[^ ^40^ ^]^ The dopant of N and B elements accompanied with the interactions between Pd and TiO_2_ support synergistically improve the electronic properties of the composite catalyst, which effectively stabilizes the active Pd species (Pd hydride) and suppresses the CO formation pathway, and thus favoring the formate production in the larger potential window than the undoped electrodes. The concurrent CO poisoning phenomenon severely affects the CO_2_‐to‐formate conversion of Pd‐based catalyst, which subsequently results in dehydration, lower formate selectivity, and decreased stability to a great extent. In this context, Lee and co‐workers proposed a system‐level strategy to effectively resolve the poisoning issue via a cyclic two‐step electrolytic process.^[^ ^41^ ^]^ The researchers alternately applied the reduction and oxidation potentials during the ECR process to selectively remove CO from the surface of Pd during the anodic step, based on the different reversibility (redox potential) of formate and CO production reactions. Therefore, the cyclic two‐step electrolysis maintains 100% current density durability and 97.8% selectivity toward formate production over 45 h testing, significantly superior to other conventional potentiostatic electrolysis.
VII) Other metallic catalysts like Co‐ and Mo‐based catalysts are also regarded as the ideal alternatives for ECR due to their earth abundance and low overpotential/free energy for *H species adsorption. Surface engineering has been explored to design highly efficient catalysts with low dimensions and large specific surface areas, which can modify the *d*‐band electronic structure of metal atoms, and thus construct suitable active sites for CO_2_ reduction. Xie's group successfully prepared atomic Co layers with high selectivity and long‐term durability under relatively low overpotential for formate production.^[^ ^42^ ^]^ The optimal ultrathin morphology and oxidation state turned an inferior catalyst into an active one for CO_2_ electroreduction, which facilitates the rate‐determining proton transfer step via the strong stabilization of HCOO* intermediate (Figure 7h–j). The Mo catalyst was optimized into a single‐atomic scale to improve the atom utilization and charge transferability, which significantly enhances the catalytic activity and formate product selectivity with abundant active sites exposed.^[^ ^43^ ^]^

#### 2.2.2 CO_2_ to CO

As important feedback of syngas, CO can be used in FT synthesis to further obtain a series of organic chemicals and intermediate products, such as, MeOH, gasoline, and diesel, etc. However, CO as a traditional CO_2_ hydrogenation product has been commonly generated from the RWGS reaction, which is an endothermic reaction activated at relatively high temperatures (>300 °C). Accordingly, converting CO_2_ to CO in an electrochemical manner is a promising alternative under ambient conditions, and CO can be easily extracted from the aqueous electrolytes for further applications, including chemicals, medicine, and the metallurgical industry.^[^ ^44^ ^]^ The electrocatalytic CO_2_‐to‐CO conversion undergoes a 2‐proton/electron transfer process which involves two elementary steps: CO_2_ molecule is first reduced and then hydrogenated to generate the surface‐adsorbed *COOH intermediate during a proton‐coupled electron transfer process.^[^ ^45^ ^]^ Afterward, the *COOH intermediate goes through the second proton/electron transfer process to convert into *CO intermediate, and then is desorbed from the catalyst surface to obtain the final product, gaseous CO. Over the past 5 years, a great deal of work has sprung up to uncover highly active and selective electrocatalysts for CO_2_‐to‐CO conversion, such as metallic nanoparticles (Au, Ag, Pd, Cu, and Zn), single atoms (Fe, Co, and Ni), and carbon‐based materials.

**Figure 8:**
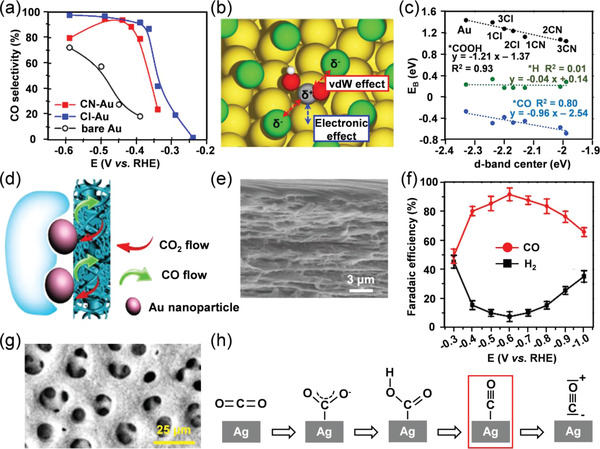
a) CO product selectivity versus applied potential of bare Au, CN‐Au, and Cl‐Au. b) The schematic illustration of van der Waals interactions and electronic effects on modified Au. c) Correlation between binding energies of reaction intermediates and *d*‐band centers up to 3/9 ML Cl and CN. Reproduced with permission.^[^ ^47^ ^]^ Copyright 2018, American Chemical Society. d) The three‐phase interface of the Au/H_2_O/CO_2_. e) SEM image of Au/nanoPE membrane. f) FE_CO_ (red curve) and FE_H2_ production (black curve) by bilayer Au/nanoPE. Reproduced with permission.^[^ ^53^ ^]^ Copyright 2018, Springer Nature Limited. g) Top‐down SEM image of the Ag foam with the primary macroporosity. h) Schematic illustration of the proposed reaction pathways toward CO_2_‐to‐CO conversion on Ag foam. Reproduced with permission.^[^ ^55^ ^]^ Copyright 2018, American Chemical Society.

**Figure 9:**
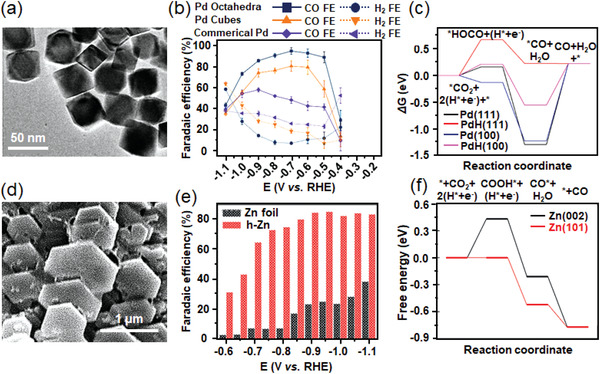
a) TEM image for Pd octahedra. b) Faradaic efficiencies of reduction products for Pd octahedra in ECR. c) DFT calculated free energy diagrams of ECR on Pd(111), Pd(100), PdH(111), and PdH(100). Reproduced with permission.^[^ ^62^ ^]^ Copyright 2019, Wiley‐VCH. d) FE‐SEM image of hexagonal Zn. e) FE_CO_ of Zn foil and hexagonal Zn at various constant potentials ranging from −0.6 to −1.1 V versus RHE. f) CO_2_ reduction pathway on Zn (002) (black solid line) or Zn (101) (red solid line) at −0.71 V versus RHE. Reproduced with permission.^[^ ^75^ ^]^ Copyright 2016, Wiley‐VCH.

I) Au‐based materials are commonly regarded as the pioneer for CO_2_ electroreduction with high selectivity of CO production (FE_CO_). However, the bulk morphology suppresses the activity of Au electrodes. Accordingly, the enhanced kinetics of the ECR in terms of nano‐Au was studied by Chen's group based on the in situ FTIR analysis,^[^ ^46^ ^]^ which directly detected the *COO^−^ and *COOH intermediates on Au surfaces. Based on the collected results, the possible reaction process of the CO_2_ reduction was described in detail. Meanwhile, some effective strategies have further been employed to focus on the optimization of nanostructural Au materials, such as surface engineering, alloying, and supported Au composites, aiming at improving the catalytic CO_2_‐to‐CO performance. The surface modification of catalysts is an effective strategy to enhance their catalytic activity due to the introduction of abundant active sites and the increased specific areas. Cho et al. modified the Au surface with additives by electroplating Au electrodes in an aqueous electrolyte with CN^−^ and Cl^−^ anions.^[^ ^47^ ^]^ Evidenced by the experimental and theoretical results, the addition of CN^−^ and Cl^−^ anions brings about van der Waals interactions and electronic effects, which significantly enhances the CO selectivity (FE_CO_ = 80% at −0.39 V vs RHE) when compared to pristine Au (FE_CO_ < 20%) (**Figure** **8**a). The van der Waals and electronic contributions are responsible for physical interactions (mainly stabilization effect) among adsorbates and chemical absorption abilities onto the metal surfaces, respectively, which promotes the stabilization of *COOH intermediate and weakens the absorption of *H species (Figure 8b,c). In addition to the anion additives, amines,^[^ ^48^ ^]^ and anchoring agent (thiol‐tethered ligands) have also been reported as effective modifiers to optimize product selectivities in ECR,^[^ ^49^ ^]^ which play similar roles in the interactions between absorbates and Au electrodes with the existence of abundant low‐coordinated sites. The construction of nanostructured surface could also bring about different coordination environments for surface atoms, which effectively adjusts their ECR performance. Narayanaru and coworkers modified the electrode roughness by changing the interfacial pH gradients, which converted the flat surface into nano and hierarchical structures.^[^ ^50^ ^]^ Such microheterogeneity of surfaces contributes to tunable product selectivity and high catalytic activity in virtue of the enriched active sites of Au. Alloying Au with other metals shifts the *d*‐band centers of Au, which changes their electronic structures and alters the interactions between intermediates and substrates. Meanwhile, alloying can also bring about geometric effects, which leads to the atomic rearrangement around the active sites and further influences the absorption capabilities of reaction intermediates onto the alloy surface. Accordingly, Au‐Pd alloys synthesized by Valenti et al.^[^ ^51^ ^]^ and Au‐Pt alloys fabricated by Ma et al.^[^ ^52^ ^]^ showed distinct catalytic activity and selectivity for CO_2_‐to‐CO conversion by varied binding strength of *COOH and *CO intermediates, which adequately demonstrated the synergistic effects of the changed electronic and geometric structures. This study would inspire more works on the synthesis of highly active and selective electrocatalysts at reduced overpotentials. Lately, some works focused on the support effect of Au catalysts, which showed great influences on the stabilization and dispersion of nanostructured Au, and provided abundant active sites and effective synergistic interactions between Au and support for enhanced ECR performance. Cui's group reported a bilayer Au/nanoporous polyethylene (nanoPE) membrane via a sputtering method,^[^ ^53^ ^]^ which exhibits an optimal FE_CO_ of ≈92% with a high geometric current density for CO production (*j*_CO_) of ≈25.5 mA cm^−2^ at −0.6 V versus RHE. The nanoPE membrane not only promotes the CO_2_ mass transport toward the reaction sites but also provides a high density of active sites at the three‐phase (Au‐H_2_O‐CO_2_) interface due to its hydrophobic property (Figure 8d–f). Jin et al. reported an Au nanocatalyst supported on the N‐doped carbon (AuNCs@CN),^[^ ^54^ ^]^ where N heteroatom enriched the surface charge density and realized a high degree of dispersion of Au. When compared to pristine Au, N‐doped carbon significantly increases the localized concentration of CO_2_ around Au active sites, which improves its selectivity toward ECR by 50% and further decreases the total cost of the catalytic system.
II) Ag‐based materials have been regarded as an ideal alternative for ECR owing to their abundant storage, low cost, and satisfying FE_CO_ compared to that of Au. Recently, numerous works have been reported to concentrate on the structure optimization of Ag with low overpotential and high FE_CO_ over 90%. Dutta et al. developed Ag‐foam catalysts via a metal deposition approach with masses of low‐coordination reaction sites,^[^ ^55^ ^]^ which showed superb FE_CO_ above 90% over a broad potential window from −0.3 to −1.2 V versus RHE (Figure 8g,h). Meanwhile, it presented long‐term durability with FE_CO_ retaining above 90% during 70 h ECR at −0.8 V versus RHE. Similarly, the sponge‐like AgCu alloy with 3D nanoporosity possessed numerous holes and interior void space,^[^ ^56^ ^]^ which led to larger ECSA and highly active local sites, thus resulting in the improved CO_2_ adsorption, electron‐proton transfer, *COOH binding capability, and *CO desorption. Other strategies have also been adopted to effectively stabilize reactants and reaction intermediates except for the structural design. For example, with the introduction of the amine functional group, Ag nanoparticles exhibited an improved selectivity for CO production (FE_CO_ = 94.2%) and effectively suppressed HER owing to its destabilization of the bonded hydrogen species.^[^ ^57^ ^]^ In addition, oxide‐derived Ag catalysts were reported to be promising candidates for high‐performance ECR. Ma et al. demonstrated an Ag catalyst derived from Ag oxides, which exhibited a decreased overpotential by more than 400 mV accompanied with a higher FE_CO_ (≈80%) than that of untreated Ag (FE_CO_ ≈ 4%) at a moderate overpotential of 0.49 V versus RHE.^[^ ^58^ ^]^ The idea was applied to another work on Ag nanoparticles,^[^ ^59^ ^]^ which incorporated stable oxygen species onto the surfaces and led to an improved CO selectivity and long‐term stability. O_2_ plasma‐treated Ag foil was also employed to construct rougher surfaces and new active sites for Ag‐based catalysts,^[^ ^60^ ^]^ which performed optimized overpotentials for CO_2_‐to‐CO conversion due to the stronger binding ability of reaction intermediates, mainly *COOH and *CO species. To further enhance the catalyst stability, SnO*_x_* species were electrodeposited on the O_2_ plasma‐treated Ag foil to construct a bimetallic system, which effectively maintained the chemical state, surface morphology, and composition of the catalysts. Similar phenomenons were further evidenced by a recent study on a highly active Ag‐alloyed Zn dendritic electrocatalyst,^[^ ^61^ ^]^ which exhibited a high CO_2_‐to‐CO selectivity of 91% and stability during 40 h testing without any obvious loss.
III) Pd‐based materials are promising catalysts for CO and formate production in ECR, which are greatly influenced by the applied potentials,^[^ ^38^ ^]^ morphologies, active sites, crystal facets, and heteroatoms doping. Recently, Chen and coworkers reported nanosized Pd octahedra with dominant Pd(111) facet,^[^ ^62^ ^]^ which showed a high CO selectivity of 95% since the binding energies of reaction intermediates (*CO and *HOCO species) were optimized on the surface of in situ formed PdH(111) under reaction conditions (**Figure** **9**a–c). Gong's group synthesized 5‐atomic‐layer hexagonal Pd nanosheets with numerous exposed edge sites,^[^ ^63^ ^]^ which reached a FE_CO_ value of 94% at −0.5 V versus RHE without any activity loss in 8 h electrolysis. Meanwhile, the DFT calculations revealed that the increased amount of edge sites exposed more active centers with a generalized coordination number of ≈5, which certainly improved the catalytic performance due to the easier *COOH formation and *CO desorption. Another work was reported by the same group,^[^ ^64^ ^]^ in which the Pd concave cubes enclosed with high‐index (310) facets showed the peak FE_CO_ value of 90.6%. Sun et al. engineered the surface of Pd nanoparticles by the introduction of CeO_2_ interfaces and Te heteroatoms,^[^ ^65^ ^]^ which could reach a FE_CO_ value over 84% and a high mass activity for CO formation of 92 mA mg_Pd_^−1^.
IV) Cu‐based materials are well‐known catalysts for high value‐added hydrocarbons production instead of CO in ECR due to their relatively high CO binding capacity. However, many efforts have also been made to adjust the absorption ability of reaction intermediates, which altered the main product from hydrocarbons to CO. To enlarge the number of active sites and atomic utilization, ultrathin Cu nanosheets^[^ ^66^ ^]^ and Cu atom‐pair catalysts^[^ ^67^ ^]^ were synthesized, which promoted the mass and electron transfer, the interaction between reactants/intermediates and Cu substrates, and intrinsic activity, thus generating optimized efficiencies, selectivities, and stability with relatively low overpotentials for CO production. In addition, some works focused on combining Cu with other metals or metal oxides to break the scaling relations and contribute to highly selective CO formation at a decreased onset potential, which was caused by the balance between *COOH and *CO binding energies on the catalyst surface. Furthermore, CuAu,^[^ ^68^ ^]^ CuPd,^[^ ^69^ ^]^ CuZn,^[^ ^70^ ^]^ CuCd,^[^ ^71^ ^]^ CuFe,^[^ ^72^ ^]^ In‐doped Cu@Cu_2_O,^[^ ^73^ ^]^ and Cu/SnO*_x_* ^[^ ^74^ ^]^ had been studied to shift the main products to CO.
V) Zn‐based materials have also been popular alternatives for ECR due to their abundant storage, low toxicity, and high CO selectivity. Woo's group reported a hierarchical hexagonal Zn catalyst,^[^ ^75^ ^]^ which realized a highly efficient (FE_CO,maximum_ = 95%) and stable performance (30 h) for CO production. The optimized (101)/(002) facet ratio effectively stabilized *COOH intermediate and suppressed HER process (Figure 9d–f). However, Zn is regarded as an active metal that can easily form surface oxide layers, which on the other hand minimizes the density of active sites and increases HER activity. Inspired by a promising “Trash to Treasure” approach, a facile anodic oxidizing method was applied to regulate ZnO species with lattice dislocation,^[^ ^76^ ^]^ which successfully converted CO_2_ to syngas (CO + H_2_) with satisfactory ratios in a wide potential window. Besides, strategies of converting ZnO to porous Zn catalysts via a pre‐treatment process^[^ ^77^ ^]^ or in situ reduction^[^ ^78^ ^]^ during the ECR process, led to an optimal stabilization capacity of the key intermediates toward CO_2_‐to‐CO conversion and therefore contributing to high FE_CO_ and *j*_CO_ values.
VI) Carbon‐based materials have been considered as highly active and stable electrocatalysts owing to their rapid ion and electron transfer capacity, as well as, chemical and thermal stability. Tremendous efforts have been made to study their applications in HER, oxygen reduction reaction, and OER, whereas they perform notorious activity toward ECR possibly due to the lack of active sites for CO_2_ activation. Accordingly, introducing heteroatoms into the carbon framework has been demonstrated to be an effective approach to enhance CO_2_ reduction activity by tuning the electronic properties of adjacent carbon atoms. Some carbon‐based materials, such as, N‐doped carbon nanotube, graphene, carbon fibers, carbon black, nanoporous carbon, and diamonds had been summarized in our previous review,^[^ ^7^ ^]^ which presented high CO selectivities and low overpotentials in ECR. Besides, Guo and co‐workers revealed the intrinsic activity of ECR on N‐doped carbon materials including graphene and CNT, through DFT and ab initio molecular dynamic calculations.^[^ ^79^ ^]^ Theoretical results calculated the barriers for CO_2_ activation and intermediates formation on different structures of carbon materials to verify the active sites and the selectivities of different products, which revealed the vital role of N species in activating CO_2_ reduction and restraining HER kinetics. Since the pyrolyzation of zeolitic imidazolate frameworks (ZIFs) has been known as a strategy to provide N‐doped carbons, Lin's group synthesized a composite material combining the pyrolyzed ZIFs with multi‐walled CNTs, which reached an extraordinary FE_CO_ value of approximately 100% at a moderate overpotential of 740 mV.^[^ ^80^ ^]^ Meanwhile, Lin's group further introduced the Fe element into the pyrolyzed ZIFs/multi‐walled CNTs hybrid and achieved a high FE_CO_ value of 97% at a decreased overpotential of only 440 mV. Similarly, Xu and co‐workers prepared a 3D N‐doped mesoporous carbon‐supported Ni catalyst, which exhibited excellent FE_CO_ of ≈98% at −0.7 V versus RHE and long‐term durability during the 25 h test.^[^ ^81^ ^]^ The enhanced performance of carbon‐based materials containing N species and transition metals (M‐N‐C) could be explained by DFT studies, which were reported in a recent review of Strasser's group.^[^ ^82^ ^]^ When compared with N‐doped carbon, the incorporation of transition metals could further enhance the reactant mass transport, electron‐proton mobility, and electronic density of the carbon framework. The doping of transition metals also played an essential role in the interplay between key intermediates and metal sites, which significantly influences the ECR performance. In particular, to realize high CO selectivity, the binding energy of *H intermediate onto the metal center should be reduced to suppress the HER side reaction. Meanwhile, to further decrease the overpotential of CO_2_‐to‐CO conversion, the absorption capability of *COOH and *CO intermediates should be optimized on metal sites.
VII) Currently, some inexpensive transition metals (e.g., Fe, Co, and Ni) with bulk morphology perform superior HER and easy CO‐poisoning phenomenon, which suppress their CO_2_ reduction to a great extent. The limited densities of active sites and poor intrinsic activity of bulk electrocatalysts hinder their ECR performance. Accordingly, atomically dispersed transition metal catalysts have recently been the popular options for CO_2_‐to‐CO conversion due to their high atomic utilization, tunable coordination structures, and suitable electronic effects. Many studies have sprung up to explore transition metal single atoms or porphyrin‐like catalysts in ECR, with a special focus on the synthetic methods and their structure‐performance relationship. N‐doped carbon frameworks have been regarded as promising supports to achieve the atomic dispersion of non‐noble metals. For example, graphene can be used as suitable support to stabilize these atomic active sites because of its specific surface area, high electronic conductivity, and controllable surface engineering. Meanwhile, heteroatom doping can further adjust the electronic structures of graphene, which promotes the strong interaction between heteroatoms and metals and thus realizes the high dispersion of metal atoms onto the graphene.

**Figure 10:**
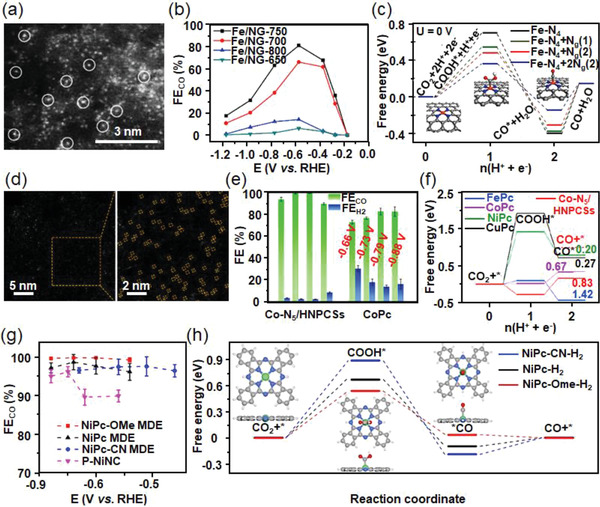
a) High magnification aberration‐corrected HAADF‐STEM image of atomic Fe/N‐doped graphene catalyst. b) Potential‐dependent FE_CO_ in ECR on Fe/N‐doped graphene single‐atom catalysts prepared at different annealing temperatures. c) Free energy diagram for ECR to CO on Fe‐N_4_ moieties supported on graphene sheets. Reproduced with permission.^[^ ^83^ ^]^ Copyright 2018, Wiley‐VCH. d) AC‐HAADF‐STEM images of Co‐N_5_/HNPCSs catalyst at different magnifications. e) The comparisons of FE_CO_ values of Co‐N_5_/HNPCSs and CoPc catalysts at various constant potentials. f) Calculated free energies of CO_2_RR on Co‐N_5_/HNPCSs and other metal phthalocyanines (FePc, CoPc, NiPc, and CuPc). Reproduced with permission.^[^ ^86^ ^]^ Copyright 2018, American Chemical Society. g) FE_CO_ values of NiPc‐OMe MDE, NiPc MDE, NiPc‐CN MDE, and P‐NiNC at different applied potentials. h) Calculated free energy diagrams of NiPc‐CN‐H_2_, NiPc‐H_2_, and NiPc‐OMe‐H_2_ for CO_2_RR at −0.11 V versus RHE. Reproduced with permission.^[^ ^92^ ^]^ Copyright 2020, Springer Nature Limited.

Tour et al. anchored atomic Fe sites on the N‐doped graphene support, which exhibited an optimal FE_CO_ value of 80% at a low overpotential (**Figure** **10**a,b).^[^ ^83^ ^]^ DFT calculations were further conducted to understand the mechanism of enhanced CO_2_‐to‐CO conversion with the existence of Fe‐N_4_ moieties. The results indicated that the Fe‐N_4_ centers effectively lowered the formation energy of *COOH intermediates and desorption ability of *CO intermediates, thus favoring the CO production (Figure 10c). Besides, N‐doped graphene support could also stabilize atomically dispersed Fe species to form the Fe‐N_5_ active sites, which formed from an additional axial pyrrolic N ligand coordinated to Fe‐N_4_ moieties.^[^ ^84^ ^]^ The DFT results revealed that Fe‐N_5_ sites could make full use of the electron density of Fe 3*d* orbitals, which suppressed the Fe‐CO *π* back‐donation effect and thus accelerated CO desorption. Herein, the Fe‐N‐C catalyst exhibited an extraordinary CO selectivity (FE_CO_ = 97%) at a low overpotential of 350 mV. To further improve the ECR performance of the Fe‐N‐C catalyst, Li's group incorporated S atoms in N‐doped microporous carbon layers, which effectively tuned the electronic structure Fe‐N active centers.^[^ ^85^ ^]^ The N, S co‐doping upshifted the Fermi energy of Fe 3*d* and increased charge density of Fe atoms on Fe‐N_4_ sites, which facilitated CO_2_ activation and strengthened the interaction between active sites and key intermediates, and thereby achieved a high FE_CO_ of 98% at a moderate overpotential of 490 mV and long‐term stability during a 30 h test without obvious activity degradation.

Atomically dispersed Co has also been a promising active site for highly effective and selective ECR. Li et al. synthesized Co single atoms supported on hollow N‐doped porous carbon spheres (HNPCSs), which were coordinated with the adjacent N atoms to form the Co‐N_5_ site (Figure 10d).^[^ ^86^ ^]^ The Co‐N_5_/HNPCSs catalyst exhibited high selectivity toward CO_2_‐to‐CO conversion with FE_CO_ above 90% over a wide potential window of −0.57 to −0.88 V versus RHE (Figure 10e). DFT studies revealed that the extraordinary performance was mainly ascribed to the atomically dispersed Co‐N_5_ sites, which effectively facilitated CO_2_ activation, *COOH intermediate formation, and *CO desorption (Figure 10f). Co‐N_4_ moieties within Co porphyrin or supported over carbon substrates have also been reported for selective CO_2_RR. The mechanism and reaction pathways of these catalysts in ECR were comprehensively investigated, aimed at gaining new insights into the efficient catalytic systems. Koper and co‐workers reported a DFT study of Co porphyrins in ECR.^[^ ^87^ ^]^ They found that the CO_2_ molecule was first activated into CO_2_^•−^ anions and bound tightly onto a Co porphyrin. Then, CO_2_^•−^ acted as a Brønsted base and obtained a proton from a water molecule, and then formed the [Co(P)‐(COOH)] intermediate. Afterward, gaseous CO was generated as the main product through a decoupled proton‐electron transfer process. Accordingly, Co porphyrins can realize high faradaic yield, turnover frequency, and low overpotential when catalyzing CO_2_ to CO. For example, a metalloporphyrin‐tetrathiafulvalene catalyst‐based covalent organic framework was prepared with Co porphyrin as building units,^[^ ^88^ ^]^ which exhibited a high FE_CO_ value of 91.3% at −0.7 V versus RHE and long‐term stability over 40 h in aqueous electrolyte. To further enhance the CO_2_ reduction performance of Co porphyrin, the catalyst could be incorporated into various organic frameworks owing to its structural flexibility. Sun et al. synthesized Co porphyrin nanotubes with a large diameter to study the curvature effect on the CO_2_RR selectivity, which preferred CO formation at a low overpotential because of its weak adsorption of *CO intermediate.^[^ ^89^ ^]^ To improve the conductivity and stability of the overall catalytic system, carbon‐based supports have been applied to immobilize the Co porphyrin forming strong *π*–*π* interactions between the porphyrins and carbon supports. Daasbjerg's group explored a facile method to directly immobilize unmodified cobalt meso‐tetraphenylporphyrin (CoTPP) onto CNT,^[^ ^90^ ^]^ which showed a high selectivity of CO production above 90% at a relatively low overpotential. In comparison, the pristine unsupported CoTPP catalyst exhibited poor product selectivity and slow reaction rate of CO_2_ conversion and required an excess high overpotential. Similarly, Han et al. covalently grafted Co porphyrins onto the CNT surface by a substitution reaction with a quite high loading of 10 wt%,^[^ ^91^ ^]^ which achieved an extraordinary FE_CO_ of 98.3% and a high current density of 25.1 mA cm^−2^ with long‐term durability at a low overpotential of 490 mV. In addition, the turnover frequency of CO production was improved by more than three times higher when compared to the physically mixed Co porphyrin/CNTs composites. The optimized configuration strengthened the catalyst‐substrate interaction, which synergistically improved the electron transfer to the intermediates and the long‐term stability of the overall catalytic system.

Ni phthalocyanines were also incorporated onto CNTs as an efficient composite for the CO_2_RR application. Liang et al. synthesized Ni phthalocyanine with ‐OMe/‐H ligands embedded on carbon nanotubes as a molecularly dispersed electrocatalyst (NiPc‐OME MDE), which achieved superior catalytic performance in terms of activity (high current density = −300 mA cm^−2^), selectivity (FE_CO_ > 99.5%), and stability (40 h) (Figure 10g).^[^ ^92^ ^]^ Observed from DFT results (Figure 10h), the energy barrier for *COOH formation is smallest in the case of NiPc‐OME, which is the rate‐determining step toward CO_2_‐to‐CO conversion, thus facilitating the activity and selectivity of CO production. Moreover, analyzed from Ni—N bond orders in NiPc molecules, the results indicated that NiPc‐OME possessed the strongest Ni—N bonds, and the existence of methoxy ligand preferred easier CO desorption, which synergistically avoided structure collapse of catalyst and made NiPc‐OME MDE an ultra‐stable CO_2_RR catalyst. Liu and co‐workers synthesized atomically dispersed Ni‐N_5_ active sites,^[^ ^93^ ^]^ which anchored the planar Ni‐N_4_ onto the N atom in the carbon matrix to form NiPc/NC catalyst. Owing to the synergistic effect of the coordinatively unsaturated Ni‐N_4_ sites and the surface pyridinic N species, NiPc/NC exhibited high FE_CO_ over 93% in a wide potential window from −0.5 to −0.8 V versus RHE, and the optimal value can up to 98% at a relatively low potential of −0.5 V versus RHE. Similarly, Ni and N co‐doped mesoporous carbon materials were fabricated by Qiu's group,^[^ ^94^ ^]^ which brought synergetic effects to enhanced CO_2_ activation, accelerated reactant, and electron transfer, as well as, a higher density of active sites in the form of Ni—N moieties in the unique ordered mesoporous structure and therefore realizing high FE_CO_ over 90% in a broad potential range from −0.75 to −1.20 V versus RHE. Lou et al. successfully decorated isolated Ni atoms onto hollow N‐rich carbon plates (Ni‐NC),^[^ ^95^ ^]^ which greatly facilitated the electron transfer and boosted the redox capability for CO_2_ reduction. The Ni atoms acted as the active sites that could adjust the energy configuration of Ni‐NC catalyst, which achieved highly‐efficient electrochemical CO_2_RR with enhanced current densities and high selectivity of nearly 100% toward CO formation in a wide potential range. Li et al. conducted a solid‐state diffusion process between the N‐doped carbon and bulk Ni metal to synthesize Ni single‐atom catalysts on self‐supported and hierarchical carbon substrate.^[^ ^96^ ^]^ The resultant catalyst exhibited a high current density of 48.66 mA cm^–2^ and a high FE_CO_ of 97% at −1.0 V versus RHE, reaching an industrial‐scale level. The aforementioned works broaden the applications of non‐noble materials into scalable, efficient, and stable catalysts.

#### 2.2.3 CO_2_ to Hydrocarbons/Oxygenates

The technologies of CO_2_‐to‐formate and CO_2_‐to‐CO conversion are comparatively mature in ECR, which can be directly used in the chemical industries. However, the synthesis of other hydrocarbons and oxygenates requires overcoming higher energy barriers for CO_2_ adsorption, activation, and the subsequent stepwise transformations of *CO intermediates, which determine the yield of C_2+_ products of ECR, as illustrated in Figure 4. The *CO dimerization is the pre‐requisite step for C_2_H_4_, CH_3_CH_2_OH, and *n*‐C_3_H_7_OH production. After the successive proton‐electron pair transfer process, the *CH_2_CHO intermediate is generated from the *CO‐CO dimerization, which is a rate‐determining step for C_2_H_4_ and C_2_H_5_OH formation. Furthermore, *n*‐C_3_H_7_OH can be produced by inserting CO into the stabilized *CH_3_CHO intermediate. C_2_H_6_ and CH_3_COO^–^ share the same *CH_2_ intermediate, which is generated from a series of hydrogenation reduction steps of *CO intermediate. Subsequently, the insertion of *CO into *CH_2_ generates CH_3_COO^–^ product, or the protonation of *CH_2_ gives *CH_3_ intermediate, which produces C_2_H_6_ via *CH_3_ dimerization. Accordingly, the formation of multi‐carbon compounds has been regarded as a complex and uncontrollable process, which is mainly attributed to the involvement of various intermediates and non‐electrochemical steps. Hence, the fabrication of highly efficient electrocatalysts for hydrocarbon and oxygenate formation deserves more research attention. Various strategies (e.g., morphology and structure design, alloying/dealloying, surface modification, and support modification) have been employed to optimize the structure of the catalysts to achieve higher formation rates and selectivities toward desired hydrocarbons and oxygenates.

І) Cu has been regarded to be the most popular alternative for ECR that can effectively produce hydrocarbons and oxygenates. Although the bulk Cu shows limited activity for ECR, the morphology and composition regulations have been demonstrated to be effective strategies to improve its catalytic performance toward ECR to hydrocarbons and oxygenates, including nanostructuring (e.g., shape, exposed facet, crystal phase, defective site, oxidation state, and particle size), alloying (Cu‐M bimetals), surface modification, and support addition.

##### Influence of Nanostructuring on Activity

Chen et al. have revealed shape‐dependent activity of Cu for ECR, which demonstrated that cube‐like and hexarhombic decahedron‐like Cu single crystals preferred C_2_H_4_ and C_2_H_5_OH production while octahedron‐like Cu produced C_1_ products.^[^ ^97^ ^]^ The different morphologies and structures determine the atomic arrangement of the Cu surface, which could alter the intermediate species and their binding energies and thus result in different reaction pathways and product distributions. Similarly, Wang's group investigated the effect of Cu shape on the ECR activity and selectivity.^[^ ^98^ ^]^ When compared to Cu nanospheres with a similar size, Cu nanocubes performed an enhanced faradaic efficiency for C_2_H_4_ formation (FE_C2H4_) of 60% and a partial current density of 144 mA cm^–2^ by gas diffusion electrodes. The improved CO_2_‐to‐C_2_H_4_ performance was mainly attributed to the exposed (100) facets on the surface of Cu nanocubes and the alkalinity effects of electrolytes. At high overpotentials (−0.6 to −0.8 V vs RHE), C—C couplings preferred to occur at (100) planes rather than (110) planes, which resulted in the higher C_2+_ selectivity on Cu nanocubes than that on Cu nanospheres. Tan et al. reported a hierarchically nanoporous Cu skeleton with large specific surface area, which not only provided the higher density of active sites but also accelerated the transport of the electron and reactant.^[^ ^99^ ^]^ Moreover, the decoration of vanadium oxide further facilitates the water dissociation and adjusts the *H adsorption energy, which lowers the energy barrier for the intermediate formation and the subsequent C—C coupling process, as evidenced by the DFT calculations. Therefore, the resultant np‐Cu@VO_2_ catalyst achieved a FE value of 30.1% for C_2_H_5_OH production with an ethanol partial current density of 16 mA cm^−2^ at −0.62 V versus RHE, which is a fourfold higher compared to the pristine nanoporous Cu.

**Figure 11:**
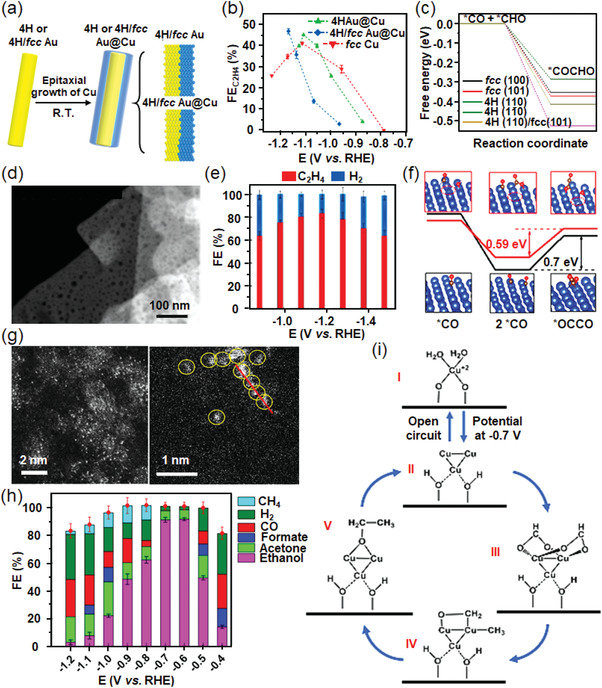
a) Epitaxial growth heterophase 4H/fcc Au@Cu core‐shell nanorod under room temperature. b) Potential‐dependent FE_C2H4_ on 4H Au@Cu, 4H/fcc Au@Cu, and fcc Cu catalysts. c) Free energy diagrams for the C—C bond formation via the coupling of *CO and *CHO on different crystal phases and surfaces of Cu. Reproduced with permission.^[^ ^100^ ^]^ Copyright 2020, American Chemical Society. d) HAADF STEM images of nano‐defective Cu NSs. e) Faradaic efficiencies of the total products at various potentials for nano‐defective Cu NSs. f) Energy diagrams and geometries of CO dimerization on Cu(111) planes of nano‐defective (red) and non‐defective (black) of Cu NSs. Adsorbed OH^–^ was considered to mimic the real environment in KOH electrolyte. Red, gray, white, and blue spheres stand for oxygen, carbon, hydrogen, and copper atoms, respectively. Reproduced with permission.^[^ ^101^ ^]^ Copyright 2020, American Chemical Society. g) HADDF‐STEM images of Cu/C‐0.4 showing the presence of isolated Cu marked by yellow circles. h) Faradaic efficiencies and the product distribution at different polarization potentials for Cu/C‐0.4 catalyst. i) The possible reaction mechanism suggested by the operando measurements on a supported Cu_3_ cluster model. Reproduced with permission.^[^ ^106^ ^]^ Copyright 2020, SpringerNature.

The crystal phase of a metallic catalyst can also affect its ECR activity to a certain extent. Most of the reported Cu catalysts for CO_2_RR are of face‐centered cubic (fcc) crystal phase, which is the thermodynamically most stable phase of Cu. However, Zhang and co‐workers recently reported unconventional 4H‐Cu and heterophase 4H/fcc Cu catalyst by using the 4H and 4H/fcc Au as templates, which exhibited enhanced activity for C_2_H_4_ formation than that of fcc Cu (**Figure** **11**a,b).^[^ ^100^ ^]^ As evidenced by theoretical calculations, C—C coupling reactions between *CO and *CHO intermediates are more energetically favored on the surface of 4H Cu and 4H/fcc Cu and thus realize higher C_2_H_4_ selectivity (Figure 11c).

Nano‐defective structure has also been constructed to facilitate the adsorption and accumulation of reaction intermediates onto the catalyst surface. Zhang et al. reported that the Cu nanosheets with abundant structure defects (nano‐defective Cu NSs) could achieve high selectivity for C_2_H_4_ production (FE_C2H4_ = 83.2%) in ECR (Figure 11d,e).^[^ ^101^ ^]^ The study demonstrated that the nano‐defective structure contributed to the enrichment of reaction intermediates and OH^–^ on the surface of the electrocatalyst, and therefore enhancing C_2_H_4_ formation via the C—C coupling reaction (Figure 11f). Twin boundary is another well‐defined and stable defective structure, which could expose truly 1D and atomic string structure, thus effectively improving the activity of the twin catalysts. Meanwhile, the exposed atoms in the twin boundary have identical chemical environments, therefore providing a simple model for theoretical exploration. Sun's group synthesized Cu twin boundaries on polished Cu electrode using a pulsed electrochemical deposition method.^[^ ^102^ ^]^ As evidenced by the intermediate experiments and DFT calculations, twin boundaries provided advantages for the stabilization of *COOH and *CHO intermediates, facilitating the conversion of absorbed *CO into CH_4_. Herein, the Cu catalyst with twin boundary atoms exhibited a local partial current density of 1294 mA cm^–2^ and an intrinsic FE_CH4_ value of 92%.

Since Cu is an active metal and can be easily oxidized to Cu^+^ species on the surface during the electrocatalytic reaction, some works attempted to understand the relationship between the ECR activity and Cu^+^ species. For example, Huang and co‐workers synthesized Cu_2_O nanoparticles enclosed with different crystal facets,^[^ ^103^ ^]^ which synergistically reached a good preference for C—C couplings. The authors ascribed the good performance to the presence of abundant low‐coordinate Cu^+^ ions on the surface and thus boosting C_2_H_4_ generation. Especially, Cu_2_O nanoparticles with (111) and (100) facets exhibited an optimal FE_C2H4_ value of 59%. Similarly, Sun's group designed a new perovskite‐type Cu_3_N nanocube with Cu(I) active species for selective CO_2_RR.^[^ ^104^ ^]^ The 25 nm Cu_3_N nanocubes showed an optimal C_2_H_4_ selectivity of 60% and stability with a 20 h testing at −1.6 V versus RHE. The experimental results and DFT calculations suggested that the (100) Cu(I) species stabilized by the Cu_3_N structure favored CO‐CHO coupling, which resulted in high selective for C_2_H_4_ formation.

To design of Cu nano‐catalysts with ultrasmall dimension has been regarded as another effective method to achieve improved catalytic activity and selectivity. To construct a unique electronic structure and maximize atom utilization, Cu‐based single‐atom catalysts have been reported for improved ECR activity. Although CO was the most common product for Cu catalysts, alcohols were also reported to be the possible products. For example, He and co‐workers synthesized isolated Cu atoms onto the carbon nanofibers in a large‐scale production method.^[^ ^105^ ^]^ The self‐supported catalyst was directly used in CO_2_RR, which realized high faradaic efficiency for methanol formation of 44%. Recently, Xu et al. reported a highly‐dispersed Cu single‐atom catalyst using amalgamated Cu‐Li as the precursor,^[^ ^106^ ^]^ which could optimize the electronic state and the stereochemistry of the active centers. The catalyst realized a single‐product faradaic efficiency of 91% at −0.7 V versus RHE toward CO_2_‐to‐ethanol conversion (Figure 11g,h). Moreover, it showed a relatively low onset potential of −0.4 V versus RHE and satisfying stability over a 16 h electrocatalytic test. The superior faradaic efficiency for ethanol formation originated from the high dispersion of Cu atoms, which benefited the reactant activation and intermediate formation as suggested by first‐principles calculations (Figure 11i).

##### Influence of Cu Alloying on Activity

**Figure 12:**
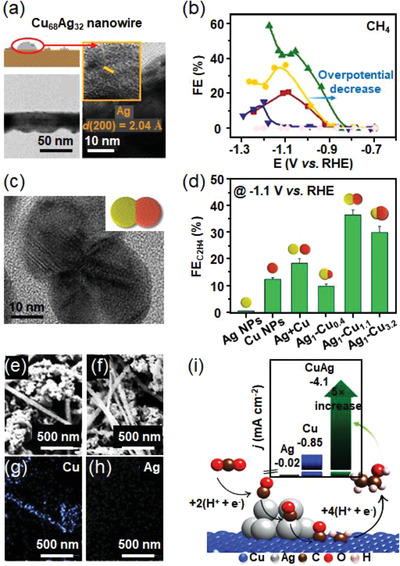
a) Diagram and TEM image of Cu and Ag for Cu_68_Ag_32_. b) Faradaic efficiency as a function of potential of CH_4_ product for Cu nanowires, and Ag‐modified Cu nanowires samples. Reproduced with permission.^[^ ^107^ ^]^ Copyright 2020, American Chemical Society. c) HRTEM images of Ag‐Cu nanodimers. d) FE_C2H4_ for Ag nanoparticles, Cu nanoparticles, and Ag‐Cu nanodimers with different Ag/Cu ratios at −1.1 V versus RHE. Reproduced with permission.^[^ ^108^ ^]^ Copyright 2019, American Chemical Society. SEM images of CuAg composite catalyst e) before and f) after 1 h ECR at −1.1 V versus RHE. The g) Cu and h) Ag EDX maps of CuAg. i) Partial current densities for ethanol production of Ag, Cu, and CuAg catalysts with a possible reaction pathway for CO_2_‐to‐ethanol conversion on CuAg sites. Reproduced with permission.^[^ ^109^ ^]^ Copyright 2020, American Chemical Society.

In addition to the structural optimization, the development of Cu‐based bimetallic catalysts has attracted wide attention for ECR application, which can break the scaling relationship and tune the interactions between catalysts and key intermediates, and thus optimize the product distributions and reach faster kinetics. Chen's group designed a model system that combined Cu nanowire with different atomic ratios of Ag as electrocatalysts for ECR.^[^ ^107^ ^]^ The study demonstrated that Cu_68_Ag_32_ nanowire exhibited the peak selectivity for methane production with a *FE*_CH4_ value of ≈60%, approximately threefold higher than that of Cu nanowires (**Figure** **12**a,b). Meanwhile, analyzed by in situ XRD, XAS, and Raman techniques, a dynamic reconstruction was detected on the surface of the catalyst, which could bring about the reoxidation of Cu^0^ and effectively stabilize the chemical state of Cu, and thus generating active species under reaction process to produce desirable products. Ag was used as an assistant to activate Cu toward the generation of multi‐carbon products according to the tandem mechanism. For example, Buonsanti et al. synthesized Ag‐Cu nanodimers where Ag nanoparticles acted as nucleation seeds for the Cu domain. Due to the tandem catalytic mechanism and unique electronic effects,^[^ ^108^ ^]^ bimetallic Ag‐Cu showed an enhanced FE_C2H4_ value by three times higher than that of pure Cu (Figure 12c,d). The Ag‐Cu nanodimers consisted of two segregated domains, which provided an optimal interface and Cu domain size for the improvement of *CO dimerization, and finally achieved the maximum C_2_H_4_ selectivity. Ag was also employed as an alternative for the ECR to ethanol formation through a closed pathway. Yeo and co‐workers mixed oxide‐derived Cu nanowires with Ag nanoparticles, which realized the maximum selectivity of ethanol formation and suppressed the C_2_H_4_ yield.^[^ ^109^ ^]^ The experimental and theoretical results indicated that the synergistic effect of Langmuir‐Hinshelwood *CO + *CH*_x_* and Cu‐Ag boundaries could contribute to the abundant CO spillover and facilitate the subsequent CO dimerization, and therefore improved the selective formation of ethanol (Figure 12e–i). Au has been regarded as another effective promoter for Cu‐based catalysts to realize satisfactory activity toward ECR. Accordingly, Sun et al. assembled Au nanoparticles on Cu nanowires with 4,4’‐bipyridine as a linker to form an Au‐bipy‐Cu composite catalyst.^[^ ^110^ ^]^ With the Au/Cu atomic ratio of 50%, the Au‐bipy‐Cu composite performed a high FE for total products of 90.6% at −0.9 V versus RHE, with a maximum FE value of 25% for methanol formation among the liquid carbonaceous product (formate, methanol, and acetate) distribution (75%). Au‐catalyzed ECR could enrich CO near Cu sites, which accelerated CO dimerization. Moreover, the addition of bipy Lewis base centers further promoted the catalytic activity of Cu catalysts since they could effectively stabilize the *CO_2_^–^ intermediates and provide abundant protons on Cu sites, synergistically resulting in high selectivities for multi‐carbon compounds production. Ternary Cu‐Au/Ag nanoframe reported by Peng et al. was also an ideal catalyst to break the linear scaling relationship of the interactions between catalyst and intermediates, and overcome the kinetic barriers of CO_2_ conversion.^[^ ^111^ ^]^ Evidenced by operando and DFT studies, Ag/Au structures played a synergistic effect for high content of CO formation (CO_2_ → *CO), while Cu was responsible for the CO—CO coupling (2*CO → *OCCO), thereby realizing a satisfying FE_C2H4_ value of 69 ± 5% with extraordinary catalytic stability and material integrity. The work emphasized the cooperative roles of tandem effect, electronic effect, and defect engineering, which offered some insights for achieving highly efficient CO_2_–to–C_2+_ compounds conversion. Owing to its low cost and non‐toxicity properties, bimetallic CuZn is an ideal alternative for ECR to realize the high activity and selectivity of carbonaceous products. Cuenya's group employed Cu_100‐_ *_x_* Zn*_x_* nanoparticles to explore the activity‐structure relationship.^[^ ^70^ ^]^ When the concentration of Zn in the range from 10% to 50%, CH_4_ was determined to the main product with a peak value of FE_CH4_ ≈70%, while HER side‐reaction was effectively suppressed. However, when the content of Zn continuously increased, the selectivity of CH_4_ decreased and CO became the main product in ECR. In virtue of operando XAFS and XPS analysis, the alloying of Cu atoms with Zn played a vital effect in the product selectivity. The variations of Cu‐Zn interaction during ECR could switch the selectivity from CH_4_ on Cu‐ZnO species to CO over CuZn alloy species. Therefore, when Cu and Zn atoms homogeneously distribute in the CuZn catalysts, CO could spill from Zn to Cu sites, thus greatly improving the formation of high value‐added products. Furthermore, Goddard's group synthesized Cu‐Bi nanoparticles, which exhibited a high FE_CH4_ value of 70.6% at −1.2 V versus RHE, ≈25 times higher than that of Cu nanoparticles.^[^ ^112^ ^]^ The experimental results indicated that the partially oxidized Cu acted as the possible active sites due to the electron withdrawal ability of Bi, which significantly lowered the formation barrier for *COH formation, and thus realized high activity and selectivity in ECR.

##### Influence of Surface Modification on Activity

Surface modification of Cu catalysts with organic additives is another effective strategy to optimize the electronic structure and alter the interactions between catalysts and intermediates and thereby facilitating the kinetic rates and product selectivity toward CO_2_RR. Wang's group designed an amino acid (zwitterionic glycine) modified Cu electrode, which stabilized the key intermediate *CHO and further enhanced the selectivity of hydrocarbons including C_2_H_4_, C_2_H_6_, and C_3_H_6_.^[^ ^113^ ^]^ Similarly, Zhang et al. introduced —NH_2_ terminated methylcarbamates onto Cu surface, which accelerated CO_2_ conversion by 17% and achieved an extraordinary FE_CH4_ value of 81.6% due to the affinity interaction between Lewis acid (—NH_3_^+^) and Lewis base (CO_2_).^[^ ^114^ ^]^ DFT results emphasized that the —NH_3_^+^ groups could stabilize *CO and *CHO intermediates onto Cu surface, which facilitated the subsequent hydrogenation reaction toward hydrocarbons production. Pyridine has also been selected as another additive to improve the catalytic activity and selectivity of Cu electrodes. For example, Agapie et al. synthesized a novel catalyst combining polycrystalline Cu electrode with N‐substituted pyridinium additives, which achieved a high selectivity for C_2_ and C_3_ hydrocarbon production, especially with a quite high ratio of C_2+_/CH_4_ (>100).^[^ ^115^ ^]^ Similar to the previous work, Agapie's group employed a simple approach to integrate a polycrystalline Cu electrode with an *N*,*N*’‐ethylene‐phenanthrolinium dibromide additive to form a composite catalyst,^[^ ^116^ ^]^ which realized the selectivities for C_2+_ products up to 70% with stable catalytic activity as well as structural integrity upon 40 h test at relatively low overpotentials. The organic additives played essential roles in the improved ECR process. In detail, the organic additives could stabilize the nanostructure on the surface of Cu, which improved C—C dimerization and suppressed HER and CH_4_ formation, thus realizing the highly efficient and durable C_2+_ chemical formation during the ECR process.

##### Influence of Supports on Activity

**Figure 13:**
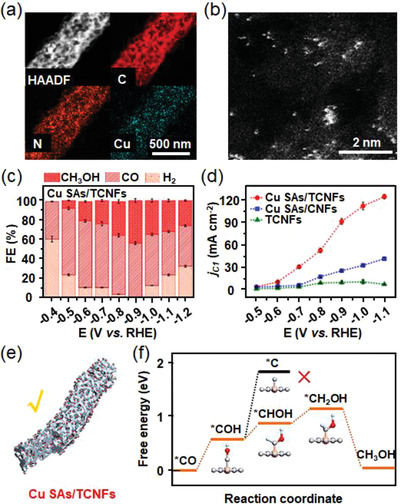
a) HADDF‐STEM image and the corresponding EDS elemental mappings, as well as, the b) AC HADDF‐STEM image of CuSAs/TCNFs. The white dots in (b) indicate the isolated Cu atoms. c) Faradaic efficiencies of all products at CuSAs/TCNFs. d) Partial current densities of CuSAs/TCNFs, CuSAs/CNFs, and TCNFs. e) Illustration of CO_2_ molecule diffusion om CuSAs/TCNFs. f) Free energies of *CO conversion to methanol on Cu‐N_4_ structure. Orange, gray, dark blue, red, and light blue spheres stand for Cu, C, N, O, and H atoms, respectively. Reproduced with permission.^[^ ^105^ ^]^ Copyright 2019, American Chemical Society.

Anchoring Cu on specific support brings about a synergistic effect for the catalytic system. First, the well‐dispersed metal catalysts can expose abundant active sites, and thus accelerate the reaction rates toward ECR. Second, the strong interaction between Cu‐based catalysts and supports can prevent the aggregation of Cu sites, which therefore significantly maintains the structure and composition of the catalysts and realizes long‐term stability. For example, N‐doped graphene,^[^ ^117^ ^]^ N‐doped carbon,^[^ ^118^ ^]^ and MoS_2_ nanoflowers^[^ ^119^ ^]^ have been selected as ideal substrates to strongly immobilize Cu nanoparticles via coordination structure or defect sites, which could stabilize the reaction intermediates and therefore improve the selectivities toward high value‐added products such as CH_4_ and ethanol at moderate overpotentials in ECR. However, the metal utilization of nanoparticles is not satisfactory, for example, only 20% of atoms are exposed on a 2 nm particle. Thereafter, dispersing Cu as isolating single atoms emerges as a flourishing approach to maximum the atom economics and improve the catalytic performance in ECR, which can also increase the stability by preventing Cu atoms from oxidation or aggregation due to the strong metal‐support interaction. However, Cu single‐atom electrocatalysts commonly undergone the two‐electron transfer process to generate CO and formate as the main products, while the further transformation into hydrocarbons or alcohols has been rarely reported so far. Accordingly, He et al. designed a facile method to realize the large‐scale synthesis of atomically dispersed Cu onto the surface of through‐hole carbon nanofibers (CuSAs/TCNFs) (**Figure** **13**a,b).^[^ ^105^ ^]^ The as‐obtained CuSAs/TCNFs catalyst reached a high faradaic efficiency for methanol production (FE_methanol_) value of 44% with an extraordinary partial current density for C_1_ chemicals of −93 mA cm^–2^ and great durability of 50 h (Figure 13c,d). DFT calculations indicated that the atomic Cu sites could stabilize *CO intermediate which could be further hydrogenated into methanol (Figure 13e,f). Noted that only C1 chemicals (i.e., methanol and CH_4_) could be produced using this catalyst in ECR. Accordingly, DFT calculations simulated the reaction pathways of CO_2_‐to‐methanol or CO_2_‐to‐CH_4_ on the surface of CuSAs/TCNFs, indicating that the *COH intermediate preferred to be hydrogenated into *CHOH (≈0.86 V) rather than *C (≈1.88 eV), which was the key intermediate for CH_4_ formation. Accordingly, the atomic Cu sites on CuSAs/TCNFs preferred methanol formation rather than CH_4_. Other supports (e.g., CeO_2_,^[^ ^120^ ^]^ TiC, and TiN^[^ ^121^ ^]^) have also been employed as ideal supports to prepare Cu‐based single‐atom catalysts by taking advantage of their oxygen vacancies or surface defect sites. The resulting electrocatalysts performed high selectivity for CH_4_ generation at quite low overpotentials, primarily because of the highly effective Cu atoms for CO_2_ activation, intermediate stabilization, and CO_2_ hydrogenation.

**Figure 14:**
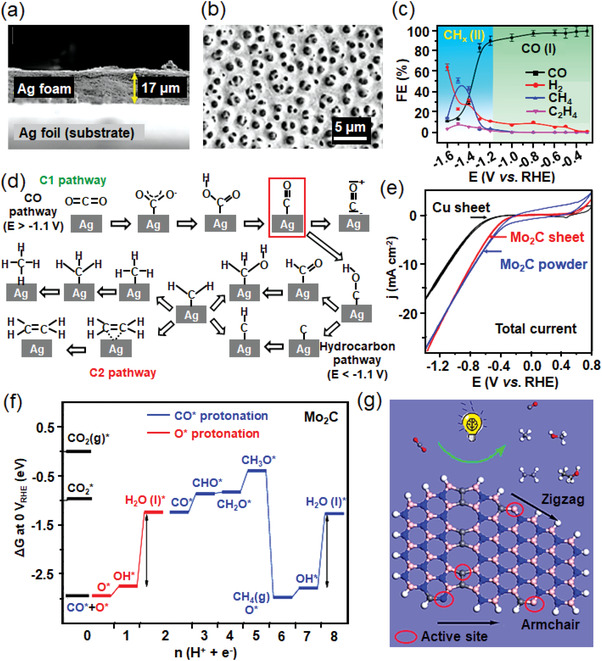
a) Side‐view and b) top‐down SEM images of the Ag foam. c) The plot of the Faradaic efficiencies as a function of applied potentials for Ag foam. d) Scheme illustration of the proposed reaction pathway of CO and hydrocarbon formations on Ag foam. Reproduced with permission.^[^ ^55^ ^]^ Copyright 2018, American Chemical Society. e) CV of Cu sheet, Mo_2_C sheet, and Mo_2_C bulk powder for ECR in 0.1 m KHCO_3_. f) Free energies of CO_2_ conversion to CH_4_ over Mo_2_C(100) surface. Reproduced with permission.^[^ ^124^ ^]^ Copyright 2016, American Chemical Society. g) Different active sites of BN for the switchable selectivities of reduction products. Reproduced with permission.^[^ ^127^ ^]^ Copyright 2018, American Chemical Society.

II) Most studies have shown that Au and Cu‐based materials are regarded as the only choices to achieve highly selective and active performance for conversion of CO_2_ to C_2+_ chemicals. Therefore, it is worthy to develop a novel efficient electrocatalyst from other metals for C_2+_ hydrocarbons and oxygenates formation. Ag used to be reported preferring CO formation, while it was employed by Broekmann's group for hydrocarbons production.^[^ ^55^ ^]^ The researchers adopted an electrodeposition method to construct a novel foam‐like Ag nanocatalyst, which created abundant low coordinated active sites because of the existence of surface porosity (**Figure** **14**a,b). The surface sites could increase *CO intermediate binding capacity and thereby increase *CO surface coverage and suppressing the HER. Furthermore, the active sites effectively lowered the kinetic energy barrier for the subsequent *CO hydrogenation in *CHO intermediate. In addition, the surface sites enabled the stabilization of *CHO species for the final hydrocarbon production. Accordingly, Ag‐foam catalyst achieved a maximum FE_CH4_ value of 51% at a higher potential of −1.5 V versus RHE, (Figure 14c), together with a maximum FE_C2H4_ value of 8.6%. The Ag‐foam catalyst designed in this work shared a common reaction mechanism with the traditional Cu‐based catalysts, which provided new insights for CO_2_‐to‐hydrocarbons/oxygenates conversion (Figure 14d). Roy et al. reported carbon‐supported PtZn alloys in the ECR study for the first time,^[^ ^122^ ^]^ which preferred methanol production. The experimental and DFT results demonstrated that PtZn alloys could facilitate CO_2_ activation and CO_2_^–^ intermediate stabilization onto the surface. Meanwhile, the weaker interaction between alloy surface and *OCH_3_ intermediate contributed to high methanol selectivity. Bocarsly designed intermetallic Ni_3_Al thin film that could produce C_1‐3_ oxygenates, including methanol and 1‐propanol at a quite low applied potential (−1.38 V vs Ag/AgCl).^[^ ^123^ ^]^ It is the first time to report the metal alloy in generating C_3_ product with the stability of the overall system more superior to that of Cu‐based systems.

Furthermore, transition‐metal carbides and chalcogenides have also attracted considerable attention for ECR due to their easy‐available property, unique electronic and geometric structures. Peterson et al. found that Mo_2_C could realize CO_2_‐to‐CH_4_ conversion at low potentials and the onset potential is only −0.55 V versus RHE,^[^ ^124^ ^]^ which is much lower than that of Cu electrocatalysts (onset potential of −0.80 V vs RHE) (Figure 14e). The main reason is that the carburizing transition metals could effectively tune the electronic structures of the catalysts and lower the free energy difference between *CO and *CHO intermediates, and thus decreasing the overpotential required for CH_4_ production (Figure 14f). Because of the unique structure, the ultrathin MoTe_2_ nanosheets were prepared by destroying the week van der Waals force between adjacent layers.^[^ ^125^ ^]^ Due to the increased specific surface area and high density of low‐coordinated surface sites, the ultrathin MoTe_2_ nanosheets performed an optimal FE_CH4_ value of 83% ± 3% and extraordinary durability of 45 h at −1.0 V versus RHE in an ionic liquid electrolyte, which could be comparable to the as‐reported Cu‐based catalysts toward CO_2_RR. The aforementioned works provide new inspirations for the conversion of CO_2_ to valuable chemicals by using other transition metals rather than metallic copper catalysts.

Recently, N‐doped carbon materials, especially N‐doped graphenes have been found to achieve the electrocatalytic reduction of CO_2_ to CH_4_ without metal sites, which process controllable structures, high stability, and easily accessible properties. Han et al. reported a graphene‐like N‐doped carbon material,^[^ ^126^ ^]^ which realized FE_CH4_ value as high as 93.5% in an ionic liquid electrolyte, and the current density was sixfold higher than that of Cu electrocatalyst at the same reaction condition. Hexagonal BN sheet with a similar structure as graphene has also attracted increasing attention in the catalysis field. Recently, Luo et al. synthesized C‐doped and line‐defect embedded BN nanoribbons for ECR,^[^ ^127^ ^]^ which could switch selectivities of different products at various active sites (Figure 14g). For example, C‐doped zigzag and armchair BN nanoribbons presented strong adsorption ability to the *CO intermediate, which promotes CH_4_ formation after easy subsequent hydrogenation of *CO. When referred to the role of edge B atoms and C dopants, they would act as dual active sites to facilitate the couplings between *CH_2_ and *CO intermediates and thereby contributing to a high selectivity for C_2_H_4_ or ethanol formation. In conclusion, Cu‐based materials, other transition metals, their alloys/carbides/chalcogenides, and metal‐free catalysts have offered more choices and opportunities for satisfactory CO_2_‐to‐hydrocarbon/oxygenate conversion with optimal activities, selectivities, and long‐term stabilities.

Concluded from above‐mentioned research work, different types of metal or non‐metal based materials might exhibit various selectivities for a specific product. Therefore, we meticulously discussed the representative catalyst types and the corresponding catalytic mechanisms in each reaction pathway part, aiming at guiding the readers to rationally design the catalysts for highly‐selective CO_2_‐to‐specific product conversion. For example, due to the high intrinsic activity in converting CO_2_ to CO, Au‐ and Ag‐based materials have been intensively studied as the model catalysts for achieving high CO yield. Furthermore, systematic overviews have been unfolded on catalysts concerning design principles, covering the aspects of nanostructuring (e.g., shape, exposed facet, crystal phase, defective site, oxidation state, and size), alloying, surface modification, and support addition, to optimize the catalytic performance of the designed catalysts. These principles can act as the exemplary embodiments for other novel material design, so as to grasp the critical factors affecting the catalytic performance. Last but not the least, the above‐mentioned contents emphasize the structure‐activity relationships of electrocatalysts in ECR mainly from the aspects of the reactant diffusion/adsorption, intermediate types and the adsorption/desorption energy on the active sites of catalysts by in situ/operando characterizations and DFT calculations. Thus, the origin of selectivity toward a specific carbonaceous product on the basis of catalysis principles is more clear. Therefore, this section could provide a better mechanism understanding and guide for the development of the next‐generation catalysts.

## 3 Photoelectrocatalysis

### 3.1 Motivation and Principles

When compared with photocatalysis, photoelectrocatalysis has more choices in semiconductors, which can better meet the requirement of suitable band position and redox potential level for CO_2_ reduction with the assistant of an external bias. When compared with electrocatalysis, photoelectrocatalysis can decrease the overpotentials toward CO_2_ reduction under light irradiation. In general, the photoelectrocatalytic CO_2_ reduction processes can be classified into two systems, that is, half‐cell and full‐cell. However, the latter has been regarded as an ideal photoelectrochemical (PEC) CO_2_ conversion, which takes place in an aqueous electrolyte to produce carbonaceous products and oxygen in photocathode and photoanode via CO_2_ reduction and H_2_O oxidation reactions, respectively. Accordingly, highly efficient materials are required to be elaborately designed to simultaneously boost CO_2_‐to‐fuels conversion and OER at a large photovoltage without any sacrificial reagents. Namely, the photoelectrode materials should possess a suitable band structure. In detail, the CB position of a photocathode semiconductor is required to be negative than the reduction potentials of CO_2_‐to‐chemicals conversion aiming at achieving CO_2_ activation and C = O bond cleavage, while the VB position of a photoanode semiconductor should be positive than the H_2_O oxidation potential. Hitherto, various electrode materials have been developed for efficient photoelectrocatalytic CO_2_ reduction, such as pristine semiconductors, heteroatom‐doped semiconductors, metal/semiconductor heterojunctions, metal complex/semiconductor hybrids, and nanostructured semiconductors, etc. These adopted strategies aim at enhancing light absorption ability, photo‐induced carrier mobility, and catalytic stability, finally realizing highly efficient photoelectrocatalytic CO_2_ reduction with satisfying solar energy conversion efficiency.

### 3.2 Plausible Routes for CO_2_ Conversion

Similar to the aforementioned techniques, the products generated from photoelectrocatalytic CO_2_ conversion can be generally divided into four categories: Formate, CO, alcohols (mainly methanol and ethanol), and some other hydrocarbons. The different photoelectrodes, applied potential, and reaction conditions can lead to distinct CO_2_ conversion pathways, therefore resulting in different products.

#### 3.2.1 CO_2_ to Formate/Formic Acid

**Figure 15:**
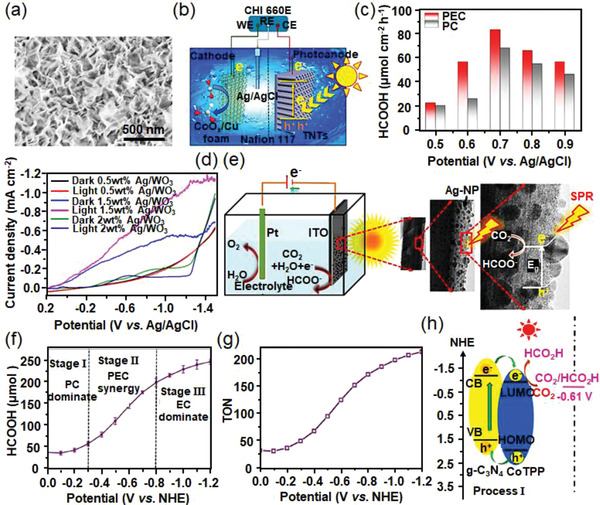
a) SEM image of CoO*_x_* nanosheets supported on Cu foam. b) Schematic illustration of photoelectrochemical CO_2_ conversion system by using CoO*_x_* nanosheets/Cu foam as photocathode and TNTs as photoanode. c) Photoelectrochemical and electrochemical CO_2_ conversion performances of CoO*_x_* nanosheets/Cu foam. Reproduced with permission.^[^ ^128^ ^]^ Copyright 2020, Elsevier B.V. d) LSV curves of Ag/WO_3_‐NR with different amounts of Ag in photoelectrochemical and electrochemical CO_2_ conversion, respectively. e) Schematic of the possible reaction mechanism of photoelectrocatalytic CO_2_‐to‐formate conversion by using Ag/WO_3_‐NR catalyst. Reproduced with permission.^[^ ^130^ ^]^ Copyright 2020, Elsevier Ltd. f) Product yield and g) TON tendency of formic acid formation under different potentials of photoelectrochemical CO_2_ conversion with Co porphyrin/g‐C_3_N_4_ catalyst. h) The proposed charges transfer pathway of Co porphyrin/g‐C_3_N_4_ catalyst toward CO_2_RR. Reproduced with permission.^[^ ^131^ ^]^ Copyright 2017, The Royal Society of Chemistry.

Li et al. designed a CoO*_x_* nanosheet‐like photocathode combined with the photoanode of TiO_2_ nanotube arrays (**Figure** **15**a),^[^ ^128^ ^]^ which achieved a peak value of FE_formic acid_ (60.9%) at a small applied potential (−0.85 V vs Ag/AgCl) in CO_2_ electroreduction. When compared with electrocatalysis, the PEC system exhibited an optimal formic acid production yield excess 80 µmol cm^–2^ h^−1^ with the assistant of solar energy and enhanced performance at the low potentials ranging from 0.5 to 0.9 V (vs Ag/AgCl), which realized efficient utilization of solar energy and high‐performance CO_2_ conversion (Figure 15b,c). To boost the photoelectrocatalytic efficiency of pure semiconductors, heteroatoms‐doping into the structure of catalysts has been demonstrated to be an effective approach to facilitate the CO_2_/intermediate activation. Herein, Yang et al. reported a Bi, S co‐doped SnO*_x_* catalyst for photoelectrocatalytic CO_2_ reduction,^[^ ^129^ ^]^ which provided abundant active sites and surface defects for formate production, and therefore achieved an ideal CO_2_ conversion process with a relatively low overpotential and high partial current density compared with pure SnO_2_ catalyst. The Bi, S co‐doped SnO*_x_* catalyst showed higher photocurrent density than that of SnO_2_, indicating that the heteroatoms doping could enhance the separation efficiency of photo‐excited electron‐hole pairs and improve the visible‐light absorption ability due to the narrower bandgap. Besides, noble metals and nanostructuring have been adopted to improve the photoelectrochemical activity of semiconductors. For example, WO_3_ has been regarded as a popular semiconductor material with good optical and conductive properties for photoelectrocatalytic reactions, while the large bandgap and the rapid recombination of electron/hole pairs largely restain the photoelectrochemical efficiency of WO_3_. Accordingly, Bal's group employed a one‐pot synthetic approach to prepare highly‐dispersed Ag embedded on WO_3_ nanorods (Ag/WO_3_‐NR).^[^ ^130^ ^]^ The synergic interface of Ag/WO_3_‐NR catalyst contributed to the highly efficient photoelectrocatalytic conversion of CO_2_ to formate. The SPR effect of Ag nanoparticles not only generated hot electro/hole pairs to activate CO_2_ hydrogenation (Figure 15d,e) but also selectively produced formate when applied external potentials. Therefore, Ag/WO_3_‐NR exhibited a significantly enhanced current density and an optimal production rate of formate (31.7 mmol h^–1^) at a given potential of −1 V versus Ag/AgCl in PEC CO_2_ reduction, which overcame the required overpotential for ECR system with the assistance of light irradiation. Chemically combining metal complexes with semiconductor photo‐absorbers has been regarded as a promising way to attract more efficient photo‐induced electrons for highly active CO_2_RR performance. Zhao and co‐workers have designed a biomimetic photoelectrocatalyst composed of Co porphyrin and g‐C_3_N_4_ nanosheet via *π*–*π* supramolecular interaction,^[^ ^131^ ^]^ which performed a satisfying formic acid production yield of 154.4 µmol during 8 h photoelectrocatalytic CO_2_ conversion with a TON of 137 and nearly 100% selectivity for liquid products at −0.6 V versus NHE with the existence of light source (Figure 15f,g). The Mott‐Schottky plot and DFT calculations demonstrated the reaction mechanism of Co porphyrin/g‐C_3_N_4_ nanosheet catalyst for PEC CO_2_ reduction as follows (Figure 15h). Under solar light irradiation, the photo‐excited electrons could transfer from g‐C_3_N_4_ nanosheet to the CB of Co porphyrin, while the holes fluxed from Co porphyrin to the VB of g‐C_3_N_4_ nanosheet, and thereby resulting in the formic acid product via the Co porphyrin‐CO_2_ adduct with abundant protons. The aforementioned studies provide constructive insights into the synthesis of efficient PEC catalysts for formic acid/formate with a high production yield. On the other hand, these studies are conducive to enhancing the performance of photoelectrocatalytic CO_2_ reduction with improved reaction kinetics and clear

#### 3.2.2 CO_2_ to CO

**Figure 16:**
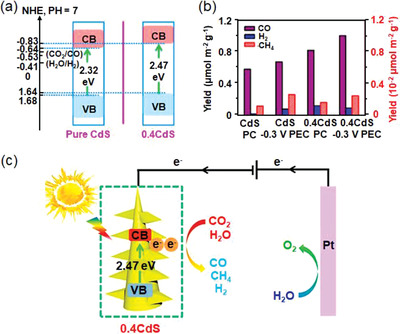
a) Bandgap structures of pure CdS and H_2_O_2_ treated CdS. b) Normalized yields of CO, H_2_, and CH_4_ generated from photoelectrochemical (left axis) and electrochemical (right axis) CO_2_ conversion by using pure CdS and H_2_O_2_ treated CdS. c) Schematic illustration of the possible mechanism of PEC CO_2_ reduction over H_2_O_2_ treated CdS under visible light illumination by applying a negative bias. Reproduced with permission.^[^ ^132^ ^]^ Copyright 2019, American Chemical Society.

Photoelectrocatalytic CO_2_ reduction to syngas is an important platform to produce CO. So far, some semiconductors (e.g., TiO_2_, WO_3_, g‐C_3_N_4_, and CdS) have realized efficient CO_2_ reduction half‐reaction during photoelectrocatalysis, among which CdS is a promising candidate owing to its suitable bandgap for solar‐light absorption and the appropriate CB position for CO_2_‐to‐fuels conversion. Accordingly, Yu et al. prepared a CdS catalyst with the presence of Cd vacancies via a facile hydrothermal approach by the addition of H_2_O_2_,^[^ ^132^ ^]^ which enhanced the separation efficiency of photo‐excited electron/hole pairs and widened the light‐response range (**Figure** **16**a). Under visible light irradiation, the H_2_O_2_ treated CdS catalyst exhibited high CO selectivity and a satisfying CO formation rate of 316 µmol g^–1^ h^–1^, which showed a 2.1‐fold higher yield than that of the pristine CdS under a negative voltage (Figure 16b). Meanwhile, the photo‐generated holes of CdS electrode would be consumed by the electrons from the external circuit during the PEC test and thereby preventing the CdS from being self‐oxidized by the photo‐induced holes and enhancing the stability of the catalytic system (Figure 16c). Additionally, other metals and metal complexes have also been adopted as cocatalysts for the selective CO_2_‐to‐CO conversion in photoelectrocatalysis. For example, Wang's group reported an Ag nanoparticle decorated p‐Si nanowire array catalyst,^[^ ^133^ ^]^ which could realize photoelectrocatalytic CO_2_ reduction to syngas with the adjustable H_2_/CO molar ratio ranging from 1 to 4. The Ag nanoparticles with the size of 8.2 nm provide a balance between *CO formation and CO adsorption, which selectively produced CO product with a FE_CO_ value of 53% with an optimal current density of 4.1 mA cm^–2^ and high faradaic efficiency for syngas formation up to 97%. Si nanowires have been regarded as highly efficient photoanodes, which possess high activity, wide light‐response range, rapid photo‐induced carrier mobility, and long‐term durability properties. Meanwhile, the CO adsorption ability could be regulated by modulating the sizes of Ag nanoparticles, which could influence the molar ratio of H_2_/CO that is suitable for the production of different downstream products, such as, paraffin, olefins, and alcohols. Kang et al. proposed a hybrid photoelectrocatalyst combining Si wafer photoanode with Co complex photocathode ([Co(tpy)_2_](PF_6_)_2_ or [Co(bpy)_3_](PF_6_)_2_) toward CO_2_‐to‐CO conversion,^[^ ^134^ ^]^ realizing a photocurrent density of 1.4 or 1.0 mA cm^–2^ with a photovoltage of 400 mV. Moreover, the FE_CO_ value could reach up to 83 or 94% in a CH_3_CN/MeOH electrolyte, which significantly improved the catalytic selectivity in photoelectrocatalytic CO_2_ reduction system. The above studies shed light on the design of highly active photoelectrocatalysts for syngas production, which will inspire more research works on efficient photoelectrocatalytic CO_2_ reduction by employing cost‐effective, environmentally friendly, and homogeneous/heterogeneous systems.

#### 3.2.3 CO_2_ to Alcohols

**Figure 17:**
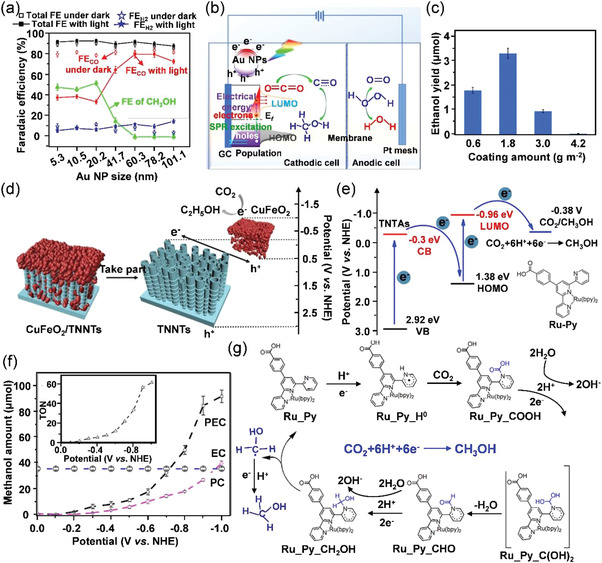
a) Faradaic efficiencies for MeOH production at different sizes Au NPs‐modified GC electrodes at −0.8 V (vs RHE) under dark and illumination with light of 520 nm and 120 mW cm^–2^. b) Schematic illustration of the proposed mechanism of SPR‐mediated CO_2_RR in photoelectrolysis by using Au NPs‐modified GC cathode. Reproduced with permission.^[^ ^135^ ^]^ Copyright 2020, American Chemical Society. c) EtOH yields of CuFeO_2_/Nb‐TNTs photocathode with different coating amounts at −0.4 V under illumination. d) Possible mechanism of photoelectrochemical CO_2_ reduction over CuFeO_2_/Nb‐TNTs. Reproduced with permission.^[^ ^137^ ^]^ Copyright 2020, Elsevier Inc. e) Energy levels positions of the Ru‐Py HOMO‐LUMO orbital and VB‐CB positions of TNTs. f) MeOH production amount and TON growth during different irradiation times for Ru‐Py/TNTs. g) Proposed reaction pathway toward photoelectrochemical CO_2_ reduction over Ru‐Py/TNTs. Reproduced with permission.^[^ ^139^ ^]^ Copyright 2017, Elsevier B.V.

Compared with formic acid/formate and syngas synthesis, alcohol synthesis from photoelectrocatalytic CO_2_ reduction has more significance and commercial importance, which can be used as an environmental‐friendly energy carrier that is compatible with the current industries. Plasmon‐mediated electrocatalysis has been regarded as a promising approach to realize highly active and selective CO_2_ conversion into higher value‐added chemicals. For example, Lu et al. found that the plasmonic Au was an ideal catalyst to generate abundant electrons with high photon energy,^[^ ^135^ ^]^ which could be easily injected into CO_2_ molecules and intermediates to activate the overall reaction system. Meanwhile, the generated photon flux would initiate C—C coupling toward high‐order product formation (**Figure** **17**a). Accordingly, plasmonic Au nanoparticles played a dual role, which could simultaneously act as a light absorber and an electrocatalyst to realize efficient photoelectrocatalytic CO_2_ reduction, and thereby exhibiting a high faradaic efficiency of 52% for MeOH formation at an applied potential of −0.8 V versus RHE under 520 nm, 120 mW cm^–2^ light irradiation (Figure 17b). By comparison, the Au catalyst could only generate CO product and no methanol was observed under the dark condition, which indicated that energetic charge carriers could form upon SPR excitation and further reduced the absorbed CO intermediate into MeOH via multi‐electron transfer process. The study brought a new sight in designing plasmonic catalysts for photoelectrochemical CO_2_ conversion systems. Cu as a promising candidate can transform CO_2_ into alcohols through electrocatalytic hydrogenation process, whereas high energy barriers and inferior faradaic efficiency hinder its development. Herein, electrocatalysis combined with photo‐excitation has been adopted in photoelectrocatalytic CO_2_ reduction by using Cu‐based catalysts. Navaee and Salimi reported a S‐doped Cu_2_O/CuO heterostructures,^[^ ^136^ ^]^ which showed enhanced activity and stability after S substitution toward photoelectrocatalytic CO_2_‐to‐methanol/acetone conversion with an increased bandgap and an appropriate overpotential, in good agreement with the CB position of as‐obtained S‐Cu_2_O/CuO nanoclusters. Besides, Zheng et al. designed an n‐type Nb‐doped TiO_2_ nanotubes arrays (TNTs) with p‐type CuFeO_2_ as a heterostructural catalyst,^[^ ^137^ ^]^ which exhibited broadened light absorption and rapid photo‐induced carrier mobility owing to its suitable bandgap and built‐in field of a p‐n junction. As a result, the CuFeO_2_/Nb‐TNTs photocathode realized a satisfying photoelectrochemical CO_2_ reduction with high selectivity toward EtOH formation and high performance of photoresponse. The optimal current efficiency of EtOH could up to be 75% at −0.4 V with an EtOH yield of 0.66 µmol h^–1^ cm^–2^ (Figure 17c,d). Except for heteroatoms doping, metals and metal complexes have been introduced in photoelectrochemical reactions. Taking TiO_2_ as an example, Luo et al. reported a Pd and rGO co‐modified TNTs catalyst,^[^ ^138^ ^]^ which was supported by a Ti wire. With light illumination, the Pd nanoparticles not only enhanced the visible‐light response region of the catalyst due to its SPR effect but also acted as the electron trapping centers. Subsequently, the photo‐induced electrons flowed to the surface of rGO layers, which functioned as an electron accumulation “pool” to reduce the absorb CO_2_ molecules. Therefore, Pd/rGO/TNTs/Ti exhibited improved production yields of MeOH (1624 nmol cm^–2^ h^–1^) and EtOH (536 nmol cm^–2^ h^–1^) under light irradiation assisted by an external potential (0.8 V bias potential) due to the synergistic effect of rGO and Pd nanoparticles. Notably, the ionization of CO_2_ to HCO_3_^–^ or CO_3_^2–^ in an aqueous electrolyte was the critical step during photoelectrocatalytic CO_2_ reduction, which was then reduced into MeOH or EtOH products. When referred to metal molecules cocatalyst, Zhao's group embedded Ru‐based complex (Ru‐Py) onto the surface of TNTs by a covalently binding approach (Figure 17e),^[^ ^139^ ^]^ which could reach an enhanced cathodic photocurrent density of 1.79 mA cm^–2^, 2.4 times higher than that of pure TNTs. Meanwhile, the system showed high selectivity for MeOH production with a FE value of 63.9%, TON of 62.6, and production yield of 84.8 µmol at −0.9 V under 8 h light irradiation (Figure 17f). On the contrary, the non‐covalent bonded system only achieved a MeOH yield of 41.3 µmol, which indicated the covalent linking played an essential role in carrier mobility and methanolization performance. The pyridyl that existed in Ru‐Py provided abundant active sites for the pyridiniumformate immediate formation, which significantly contributed to the highly selective MeOH production (Figure 17g). Therefore, the aforementioned studies provided meaningful methodologies to optimize the design strategies of photocathodes for alcohol synthesis toward photoelectrochemical CO_2_ reduction, which have made important significance in carbon resource recycling with the efficient utilization of solar energy and renewable electricity.

#### 3.2.4 CO_2_ to C_2+_ Hydrocarbons/Oxygenates

**Figure 18:**
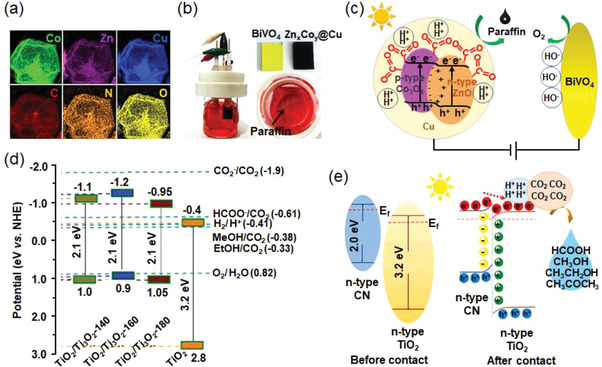
a) EDS elemental mapping images of C/N co‐doped Zn*_x_*:Co*_y_* @Cu photocathode catalyst. b) Photoelectrodes, photoelectrochemical cells, and paraffin products floated onto the surface of the electrolyte after the reaction. c) Proposed reaction mechanism of photoelectrocatalytic CO_2_‐to‐paraffin conversion by using C/N co‐doped Zn*_x_*:Co*_y_* @Cu photocathode. Reproduced with permission.^[^ ^140^ ^]^ Copyright 2019, The Author(s). d) Band positions of N‐TiO_2_/Ti_3_C_2_ photocathode and the redox potentials of CO_2_ reduction at pH 7. Reproduced with permission.^[^ ^142^ ^]^ Copyright 2018, Elsevier Ltd. e) The proposed carriers transfer mechanism of polypyrrole/TiO_2_ heterostructural catalyst during photoelectrochemical CO_2_ reduction. Reproduced with permission.^[^ ^143^ ^]^ Copyright 2020, Elsevier B.V.

As an artificial photosynthesis process, the photoelectrocatalytic CO_2_ reduction could transform CO_2_ and H_2_O into hydrocarbons and O_2_. However, the product distribution is broad and has been usually C_1_ and C_2_ chemicals, such as formic acid/formate, methanol, ethanol, and CO. Accordingly, multi‐carbon compounds have been regarded as higher value‐added products, which provide promising pathways to complete a carbon‐neutral cycle. However, there remain some challenges due to the inferior activity and poor selectivity for C—C coupling reactions. Therefore, 3D semiconductor heterostructures with abundant active sites have been designed as photocathodes to facilitate the C—C coupling pathway toward CO_2_ reduction, aiming to improve the faradaic yields of multi‐carbon hydrocarbons. Jing's group fabricated a 3D C/N co‐doped Zn*_x_*:Co*_y_* @Cu (**Figure** **18**a) p‐n heterojunction photocathode,^[^ ^140^ ^]^ which showed an optimal rate of 325µg h^–1^ toward paraffin production at −0.4 V versus SCE and apparent moderate quantum efficiency (1.95%) of the photoelectrochemical cell (Figure 18b). The as‐designed C/N‐Zn*_x_*:Co*_y_* @Cu catalyst showed an improved separation efficiency of photoinduced electron‐hole pairs due to the p‐n heterojunctions. Moreover, heteroatom doping facilitated the absorption ability and activation capability of CO_2_ molecules. Furthermore, Cu realized the significant FE for hydrocarbons and oxygenates due to its vital role in C—C coupling steps (Figure 18c). Except for metallic Cu, noble metals like Pd and Pt have also been employed as cocatalysts for efficient photoelectrocatalytic CO_2_ reduction toward hydrocarbons production. For example, Pt/graphene deposited on Cu foam was used as a 3D binder‐free photoelectrode,^[^ ^141^ ^]^ which could enhance the CO_2_ reduction performance and promote C_1_ products to higher value‐added carbonaceous compounds (e.g., acetic acid, propionic acid, and alcohols). Moreover, the carbon atom conversion rate of CO_2_ reduction increased to 5040 nmol h^−1^ cm^–2^ at a voltage of 2 V (anode served as a reference electrode) together with an enhanced current density, which was 110 times higher than that in dark. Herein, the 3D Pt/graphene/Cu foam catalyst with high porosity provided abundant active sites for CO_2_ activation and *CO dimerization for enhanced multi‐carbon hydrocarbons synthesis. In another study, Pd/N‐TiO_2_/Ti_3_C_2_ photocathode exhibited a peak apparent quantum efficiency of artificial photosynthesis cell of 1.78% at −1.0 V (anode served as a reference electrode) with a satisfying evolution rate (36.8 mm h^–1^ g^–1^) of total hydrocarbon (formate, methanol, and ethanol) formation (Figure 18d).^[^ ^142^ ^]^ Recently, polypyrrole has been decorated on TiO_2_ surface through an in situ oxidizing polymerization and temperature‐controlled pyrolysis by Jing et al, which could serve as a molecular cocatalyst for PEC CO_2_ reduction.^[^ ^143^ ^]^ The resulting n‐n heterojunctions (Figure 18e) achieved the highest selectivity (71.4%) for C_2+_ hydrocarbons (alcohols, acetic acids, and acetone) production under light irradiation at −1.2 V (anode served as a reference electrode), since the pyridine‐N sites and abundant defects prolonged the lifetime of photo‐induced carriers and facilitated the selectivity toward C—C coupling pathway. Under the photoelectrochemical conditions, polypyrrole/TiO_2_ catalyst could produce photo‐excited electrons and holes through a built‐in electric field. Due to the differences in the redox potentials of the two components, the electrons from the CB position of polypyrrole would flux to the CB of TiO_2_, while holes were neutralized by the external electrons in the circuit. Finally, the absorbed CO_2_ molecules were reduced to different C_2+_ hydrocarbons via C—C coupling reactions. In conclusion, the delicate design of heterojunctions, cocatalyst addition, and hierarchical nanostructuring can be regarded as promising approaches for selective hydrocarbons synthesis, which opened up new horizons for the fabrication of highly active photoelectrocatalysts toward artificial photosynthesis application.

## 4 Design of Flow Cell Configurations

**Figure 19:**
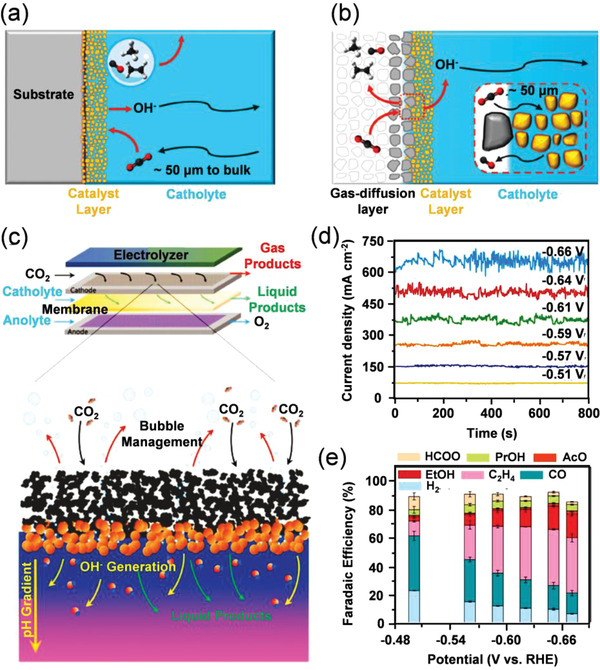
Cell views for a) an H‐cell and b) a flowing cell configuration. Reproduced with permission.^[^ ^145^ ^]^ Copyright 2019, The Royal Society of Chemistry. c) A schematic illustration of the microfluidic flow cell. d) The *I*–*T* curves and e) faradaic efficiencies of C_2+_ products at various potentials for ECR on porous Cu in 1 m KOH electrolyte using a flow cell. Reproduced with permission.^[^ ^147^ ^]^ Copyright 2018, Wiley‐VCH.

Nowadays, numerous attentions has concentrated on the overall system to extend the laboratory‐scale operation of (photo‐)electrochemical CO_2_ reduction to industrialized application, which comprehensively considers the performance of current densities, selectivities, and energy efficiencies. Except for the development of rationally‐designed catalysts, promising strategies in electrode/reactor design have remained bottlenecks that require more effort.^[^ ^144^ ^]^ Accordingly, rationally‐designed flow cell configurations have been employed to convert CO_2_ molecules to high value‐added chemicals and fuels. Schematic illustrations of H‐type and flow cells are illustrated in **Figure** **19**a,b to compare their differences.^[^ ^145^ ^]^ In an H‐type cell, only CO_2_ molecules near the working electrode can diffuse and take participate in the reduction reaction, and thereby the current density is restricted by the limited solubility and diffusion rate of the reactant. Hence, the sluggish reaction kinetics require large overpotentials to drive CO_2_RR, and the current density is usually unsatisfactory (<30 mA cm^−2^). The relatively small current density is far from the requirements for the practical industrial‐scale implementation, of which the current density should achieve up to hundreds of mA cm^–2^ and even 1 A cm^–2^. In comparison, the flow cell with gas‐diffusion electrodes (GDEs) has been regarded as an effective configuration, which circulates the electrolyte in the overall system. This not only facilitates the reaction kinetic but also stabilizes the local reaction environment as follows. First, the GDEs provide sufficient interaction between the solid catalyst, electrolyte, and CO_2_, which effectively improve the CO_2_ solubility and diffusion, and thereby enhancing the current density. Second, strong alkaline electrolytes (e.g., 1–10 m KOH) can be used in the flow cells, which significantly increases the pH value of electrolytes, thus enhancing the selectivities of carbonaceous products for CO_2_RR and suppressing the competing HER. Moreover, the use of KOH electrolyte can decrease the solution resistance, thus further improving the energy conversion efficiency of the overall system.

Recently, many representative works have sprung up associated with the applications of flow cell configurations for EC and PEC CO_2_ conversion. For example, Strasser's group loaded Ni‐N‐C catalyst onto GDEs to create a three‐phase interface in a flow cell toward electrochemical CO_2_‐to‐CO conversion.^[^ ^146^ ^]^ Compared to the H‐type cell, this large‐scale CO_2_ electrolyzer cell could be conducted at industrial current densities ≈700 mA cm^−2^. A stable FE_CO_ value of 85% was achieved for a 20‐h long‐term test with a large *j*_CO_ up to 200 mA cm^−2^. Such outstanding activity provides tremendous potential for the commercialization of the electrochemical CO_2_RR technique toward highly‐selective C_1_ chemical production. Besides CO_2_‐to‐C_1_ conversion, Cu‐based electrocatalysts have been employed in microfluidic CO_2_ flow cells to realize highly‐efficient C_2+_ chemicals production at industrially relevant rates. Jiao et al. reported the design of a highly porous Cu catalyst, which could realize an optimized selectivity of ≈62% toward C_2+_ chemicals with a high current density of 653 mA cm^−2^ at a moderate applied potential of −0.67 V versus RHE (Figure 19c–e).^[^ ^147^ ^]^ For PEC CO_2_ conversion, the flow cell configuration also contributes significantly to improving the photocurrent density and product selectivity. For example, Lu et al. designed a membrane cathode assembly with Ag nanocubes as the electrocatalyst, which exhibited higher CO_2_ solubility, better reactant diffusion, and inferior HER selectivity than that of the traditional CO_2_ conversion process conducted in an H‐type cell.^[^ ^148^ ^]^ Accordingly, the overall system achieved high electricity‐to‐chemicals efficiency of 92.1%. The photon‐to‐chemical energy efficiency reached 0.16% at the voltage of 1.2 V in a CO_2_‐fed gas system.

Besides flow cells, the membrane electrode assembly (MEA) electrolyzer has gradually developed as another intelligent device for CO_2_RR, which is composed of a cathode, anode, and polymer electrolyte membrane. The electrocatalysts are directly coated on the two sides of the membrane or loaded on the GDEs, and then pressed to sandwich the membrane. Note that the membrane plays a significant role in MEA cells, exhibiting high ionic conductivity and high chemical stability. Accordingly, the MEA electrolyzers have been successfully employed in ECR applications to produce high value‐added carbonaceous products (e.g., CO, formate, and ethylene).^[^ ^15^ ^]^

## 5 Conclusions and Perspectives

The gradual increase of atmospheric CO_2_ concentration has caused serious global warming, energy, and environmental crises. Accordingly, researchers have made tremendous efforts in CO_2_ emission reduction during past decades. Especially, CO_2_ conversion through chemical reaction path has emerged as a promising approach for the highly‐efficient CO_2_ utilization and upgradation. Thereinto, numerous technologies have been adopted to improve the CO_2_‐to‐chemicals efficiency, containing chemical reforming, thermocatalysis, photocatalysis, electrocatalysis, etc. Among which, electrocatalysis has been regarded as an advisable technology that can realize efficient CO_2_ reduction in a clean and renewable manner driven by electricity. In comparison, photoelectrocatalysis as an intelligent technology can combine solar and electronic energy as an integral whole, which not only modulates product selectivity in CO_2_ reduction by semiconductors with suitable band structure but also decreases the overpotentials with the assistance of solar energy compared to pure ECR. Meanwhile, the applied potentials indeed accelerate the separation of photo‐generated hole/electron pairs to achieve long‐term durability. Since multitudinous significant work has been continuously published regarding these fields, this review provides a timely and comprehensive overview of the state‐of‐the‐art of electro‐ and photoelectrocatalytic CO_2_ conversion into fuels and value‐added chemical feedstocks, such as formate, CO, CH_4,_ alcohols, and C_2+_ hydrocarbons/oxygenates in the past 5 years. On account of the well‐designed catalysts playing a significant role in the promoted development of electro‐ and photoelectroreduction of CO_2_, this review pays special attention to the recent development of rational designing strategies for heterogeneous catalysts toward highly‐efficient CO_2_ conversion. The reaction pathways and catalytic mechanism toward different carbonaceous products via these technologies have been briefly discussed based on in situ/operando analysis and DFT calculations, which can provide a thorough understanding of key factors about rate‐determining steps and the adsorption or formation of reactants/intermediates, and thus realizing optimal CO_2_ reduction activity and high selectivity. Accordingly, when referred to the similarities between ECR and PEC CO_2_ conversion, both techniques can effectively control the reduction products by modulating the reaction conditions (e.g., given potentials, band structures, reaction temperature). Meanwhile, both systems can be conducted in a clean and green manner driven by renewable energy sources (i.e., sunlight and electricity), and of which chemical consumption can be minimized to H_2_O. Furthermore, the (photo‐)electrochemical reaction setup is modular and compact for scale‐up application. However, when referring to the differences that exist between ECR and PEC CO_2_ conversion, the product selectivities in ECR mainly depend on the energy of electrons (namely, applied potentials) by tuning the linear‐scaling relationship of CO_2_‐reduced intermediates binding energies onto the surface of catalysts. Concerning PEC CO_2_ conversion, the electrode materials must be comprised of semiconductors. The product selectivities are not only affected by applied potentials, but also the energy of photoexcited electrons, which is determined by the CB edges of the semiconductor photoelectrodes.

However, the advancements in electro‐ and photoelectrochemical CO_2_ transformations are still not satisfactory in terms of energy efficiency and technical and economic viability for practical application. There are still some challenges and perspectives are referred to as the following points. 1) As for catalysts, cost‐effective materials and facile synthetic methodologies should be explored for large‐scale production. Meanwhile, the rational design of nanostructured catalysts for highly selective toward one specific product is of great importance. In addition, the long‐term durability of catalysts is regarded as another essential parameter that determines the practicability of catalytic systems in a practical device. In terms of photoelectrochemical CO_2_ conversion, more efforts are required to be paid into novel catalysts (e.g., semiconductor photo‐absorbers/catalysts, surface cocatalysts) to realize high solar‐to‐fuel efficiency, which should not only provide a narrow bandgap to adsorb a wide spectral range of solar light, but also perform efficient CO_2_ reduction with high selectivity and activity when given the external potentials. 2) As for reactor configurations, an H‐type electrolytic cell with an ion‐exchange membrane has been widely used in CO_2_ electro‐ and photoelectroreduction. However, to meet the requirements of industrial‐scale, ultra‐high current density, and long‐term stability should be achieved to make great progress in the high yields of valuable fuels and chemicals, providing more possibilities for future practical application. Therefore, the ration design of continuous flow cells and microfluidic have been paid much effort to reach high activity in recent years. The GDEs, flow cell, and MEA electrolyzers can effectively adjust the local environment (e.g., pH value of electrolyte) and reaction conditions (e.g., CO_2_ concentration) of CO_2_RR system, which can not only improve the reactant flow rates, but also achieve higher partial current densities, and thereby promoting CO_2_ conversion rates, efficiencies and selectivities. For example, GDEs provide a porous catalyst layer along with diffusion media to accelerate the reactant transport and distribution, aiming at achieving high current density and low transport losses. Moreover, the pH value of the employed electrolyte is an important factor in influencing CO_2_RR activity and selectivity. Especially, enhanced activity toward high value‐added C_2+_ products is favored at high pH values in the flow cell, which can hardly be achieved in a conventional H‐type system. Furthermore, flow cells and MEA provide convenience for the separation of different reduction products. The above‐mentioned advantages facilitate the large‐scale employment of CO_2_RR at industrial level. In addition, in the PEC CO_2_ reduction field, full cell and tandem cell (photovoltaic cell‐PEC reactor) has been designed with a suitable semiconductor as a photoanode for water oxidation, and simultaneously accelerate electron mobility for CO_2_‐to‐chemicals reduction, facilitating the overall solar energy conversion efficiency, and thus realizes the long‐term goal of 10% solar‐to‐fuel efficiency. 3) As for the measurement of FE and solar‐to‐fuel efficiency of EC and PEC CO_2_ reduction, a standardized metrology method should be conducted to better compare the results from different research groups, mainly concerning illumination condition, selected electrolyte and reactors, CO_2_ feeding, and product analysis methods, etc. 4) As for mechanism investigation, CO_2_ conversion processes can be explored with the assistance of advanced analysis techniques, such as, operando spectroscopy (e.g., in situ XPS, IR, and EXAFS, etc.), and computational analysis. For example, theorectical calculations has recently been regarded as an essential method for catalyst screening, *d*‐band center prediction, and Gibbs free energy trends. The DFT calculations and molecular dynamics simulations provide valuable information with existing data to conduct more sophisticated methods for holding great promise in fundamental studies of catalysis, and thereby, further pushing the development and prosperity of (photo‐)electrochemical CO_2_RR areas. Accordingly, the rate‐determining steps, the adsorption behavior of intermediates, kinetic energy barriers, and thermodynamics limits would be dealt with, which reveals the key factors for improving the activity and selectivity of catalysts. Accordingly, the combination of experimental results and theoretical studies can figure out the close relationship between catalysts surface and complicated reaction networks.

In conclusion, the comprehensive summary of fundamentals, well‐designed catalyst strategies, CO_2_RR testing, and in‐depth mechanism exploration of electro‐ and photoelectrochemical CO_2_ conversion in this review will attract wide interest from both the academic and industrial communities concentrating on material science, heterogeneous catalysis, green chemistry, as well as, energy and environmental catalysis.

## Conflict of Interest

The authors declare no conflict of interest.

## References

[1] P. E. Thornton , J. F. Lamarque , N. A. Rosenbloom , N. M. Mahowald , Global Biogeochem. Cycles 2007, 21, GB4018.

[2] J. Artz , T. E. Müller , K. Thenert , J. Kleinekorte , R. Meys , A. Sternberg , A. Bardow , W. Leitner , Chem. Rev. 2017, 118, 434. 29220170 10.1021/acs.chemrev.7b00435

[3] S. De , A. Dokania , A. Ramirez , J. Gascon , ACS Catal. 2020, 10, 14147.

[4] a) J. Qiao , Y. Liu , F. Hong , J. Zhang , Chem. Soc. Rev. 2014, 43, 631; 24186433 10.1039/c3cs60323g

[5] Y. Y. Birdja , E. Pérez‐Gallent , M. C. Figueiredo , A. J. Göttle , F. Calle‐Vallejo , M. T. M. Koper , Nat. Energy 2019, 4, 732.

[6] a) J. H. Wu , Y. Huang , W. Ye , Y. G. Li , Adv. Sci. 2017, 4, 1700194; 10.1002/advs.201700194 PMC5700640 29201614

[7] W. Zhang , Y. Hu , L. Ma , G. Zhu , Y. Wang , X. Xue , R. Chen , S. Yang , Z. Jin , Adv. Sci. 2018, 5, 1700275. 10.1002/advs.201700275 PMC5770696 29375961

[8] Y. Zheng , A. Vasileff , X. Zhou , Y. Jiao , M. Jaroniec , S. Z. Qiao , J. Am. Chem. Soc. 2019, 141, 7646. 30986349 10.1021/jacs.9b02124

[9] a) D. Gao , R. M. Arán‐Ais , H. S. Jeon , B. R. Cuenya , Nat. Catal. 2019, 2, 198;

[10] S. Xu , E. A. Carter , Chem. Rev. 2018, 119, 6631. 30561988 10.1021/acs.chemrev.8b00481

[11] G. Zhao , X. Huang , X. Wang , X. Wang , J. Mater. Chem. A 2017, 5, 21625.

[12] a) L. Zhang , Z. J. Zhao , T. Wang , J. Gong , Chem. Soc. Rev. 2018, 47, 5423; 29799046 10.1039/c8cs00016f

[13] H. Pang , T. Masuda , J. Ye , Chem. ‐ Asian J. 2018, 13, 127. 29193762 10.1002/asia.201701596

[14] V. Kumaravel , J. Bartlett , S. C. Pillai , ACS Energy Lett. 2020, 5, 486.

[15] N. Han , P. Ding , L. He , Y. Li , Y. Li , Adv. Energy Mater. 2019, 10, 1902338.

[16] L. R. L. Ting , R. Carcía‐Muelas , A. J. Martín , F. L. P. Veenstra , S. T. Chen , Y. J. Peng , E. Y. X. Per , S. Pablo‐García , N. López , J. Pérez‐Ramírez , B. S. Yeo , Angew. Chem., Int. Ed. 2020, 59, 21072. 10.1002/anie.202008289 PMC7693243 32706141

[17] N. Han , Y. Wang , J. Deng , J. Zhou , Y. Wu , H. Yang , P. Ding , Y. Li , J. Mater. Chem. A 2019, 7, 1267.

[18] J. He , X. Liu , H. Liu , Z. Zhao , Y. Ding , J. Luo , J. Catal. 2018, 364, 125.

[19] S. Liu , J. Xiao , X. F. Lu , J. Wang , X. Wang , X. W. Lou , Angew. Chem., Int. Ed. 2019, 58, 8499. 10.1002/anie.201903613 30974035

[20] L. Li , Z. J. Zhao , C. Hu , P. Yang , X. Yuan , Y. Wang , L. Zhang , L. Moskaleva , J. Gong , ACS Energy Lett. 2020, 5, 552.

[21] R. Daiyan , E. C. Lovell , N. M. Bedford , W. H. Saputera , K. H. Wu , S. Lim , J. Horlyck , Y. H. Ng , X. Lu , R. Amal , Adv. Sci. 2019, 6, 1900678. 10.1002/advs.201900678 PMC6755522 31559127

[22] Z. Chen , T. Fan , Y.‐Q. Zhang , J. Xiao , M. Gao , N. Duan , J. Zhang , J. Li , Q. Liu , X. Yi , J. L. Luo , Appl. Catal., B 2020, 261, 118243.

[23] P. Deng , H. Wang , R. Qi , J. Zhu , S. Chen , F. Yang , L. Zhou , K. Qi , H. Liu , B. Y. Xia , ACS Catal. 2019, 10, 743.

[24] C. C. Miao , G. Q. Yuan , ChemElectroChem 2018, 5, 3741.

[25] J. H. Koh , D. H. Won , T. Eom , N. K. Kim , K. D. Jung , H. Kim , Y. J. Hwang , B. K. Min , ACS Catal. 2017, 7, 5071.

[26] a) N. Han , Y. Wang , H. Yang , J. Deng , J. Wu , Y. Li , Y. Li , Nat. Commun. 2018, 9, 1320; 29615621 10.1038/s41467-018-03712-z PMC5882965

[27] D. Wu , G. Huo , W. Chen , X. Z. Fu , J. L. Luo , Appl. Catal., B 2020, 271, 118957.

[28] Y. Hori , H. Wakebe , T. Tsukamoto , O. Koga , Electrochim. Acta 1994, 39, 1833.

[29] Z. Xia , M. Freeman , D. Zhang , B. Yang , L. Lei , Z. Li , Y. Hou , ChemElectroChem 2018, 5, 215.

[30] Z. Zhang , F. Ahmad , W. Zhao , W. Yan , W. Zhang , H. Huang , C. Ma , J. Zeng , Nano Lett. 2019, 19, 4029. 31136185 10.1021/acs.nanolett.9b01393

[31] K. Mou , Z. Chen , S. Yao , L. Liu , Electrochim. Acta 2018, 289, 65.

[32] C. H. Lee , M. W. Kanan , ACS Catal. 2014, 5, 465.

[33] N. Zouaoui , B. D. Ossonon , M. Fan , D. Mayilukila , S. Garbarino , G. de Silveira , G. A. Botton , D. Guay , A. C. Tavares , J. Mater. Chem. A 2019, 7, 11272.

[34] Y. Xing , M. Cui , P. Fan , J. Ren , C. Zhang , N. Li , X. Wen , X. Ji , Mater. Chem. Phys. 2019, 237, 121826.

[35] F. Li , M. Xue , J. Li , X. Ma , L. Chen , X. Zhang , D. R. MacFarlane , J. Zhang , Angew. Chem., Int. Ed. 2017, 56, 14718. 10.1002/anie.201710038 28971548

[36] D. Gao , H. Zhou , F. Cai , J. Wang , G. Wang , X. Bao , ACS Catal. 2018, 8, 1510.

[37] A. Klinkova , P. De Luna , C. T. Dinh , O. Voznyy , E. M. Larin , E. Kumacheva , E. H. Sargent , ACS Catal. 2016, 6, 8115.

[38] D. Gao , H. Zhou , F. Cai , D. Wang , Y. Hu , B. Jiang , W.‐B. Cai , X. Chen , R. Si , F. Yang , S. Miao , J. Wang , G. Wang , X. Bao , Nano Res. 2017, 10, 2181.

[39] B. Jiang , X. G. Zhang , K. Jiang , D. Y. Wu , W. B. Cai , J. Am. Chem. Soc. 2018, 140, 2880. 29409320 10.1021/jacs.7b12506

[40] B. Bai , Q. Chen , X. Zhao , D. Zhuo , Z. Xu , Z. Wang , M. Wu , H. Tan , S. Peng , G. Guo , ChemistrySelect 2019, 4, 8626.

[41] C. W. Lee , N. H. Cho , K. T. Nam , Y. J. Hwang , B. K. Min , Nat. Commun. 2019, 10, 3919. 31477719 10.1038/s41467-019-11903-5 PMC6718411

[42] a) S. Gao , Y. Lin , X. Jiao , Y. Sun , Q. Luo , W. Zhang , D. Li , J. Yang , Y. Xie , Nature 2016, 529, 68; 26738592 10.1038/nature16455

[43] P. Huang , M. Cheng , H. Zhang , M. Zuo , C. Xiao , Y. Xie , Nano Energy 2019, 61, 428.

[44] T. Zheng , K. Jiang , H. Wang , Adv. Mater. 2018, 30, 1802066. 10.1002/adma.201802066 30129273

[45] N. J. Firet , W. A. Smith , ACS Catal. 2016, 7, 606.

[46] S. Chen , A. Chen , J. Phys. Chem. C 2019, 123, 23898.

[47] M. Cho , J. T. Song , S. Back , Y. Jung , J. Oh , ACS Catal. 2018, 8, 1178.

[48] Y. Zhao , C. Wang , Y. Liu , D. R. MacFarlane , G. G. Wallace , Adv. Energy Mater. 2018, 8, 1801400.

[49] Y. Fang , J. C. Flake , J. Am. Chem. Soc. 2017, 139, 3399. 28182409 10.1021/jacs.6b11023

[50] S. Narayanaru , J. Chinnaiah , K. L. Phani , F. Scholz , Electrochim. Acta 2018, 264, 269.

[51] M. Valenti , N. P. Prasad , R. Kas , D. Bohra , M. Ma , V. Balasubramanian , L. Chu , S. Gimenez , J. Bisquert , B. Dam , W. A. Smith , ACS Catal. 2019, 9, 3527.

[52] M. Ma , H. A. Hansen , M. Valenti , Z. Wang , A. Cao , M. Dong , W. A. Smith , Nano Energy 2017, 42, 51.

[53] J. Li , G. Chen , Y. Zhu , Z. Liang , A. Pei , C. L. Wu , H. Wang , H. R. Lee , K. Liu , S. Chu , Y. Cui , Nat. Catal. 2018, 1, 592.

[54] L. Jin , B. Liu , P. Wang , H. Yao , L. A. Achola , P. Kerns , A. Lopes , Y. Yang , J. Ho , A. Moewes , Y. Pei , J. He , Nanoscale 2018, 10, 14678. 30039128 10.1039/c8nr04322a

[55] A. Dutta , C. E. Morstein , M. Rahaman , A. Cedeño López , P. Broekmann , ACS Catal. 2018, 8, 8357.

[56] W. Zhang , C. Xu , Y. Hu , S. Yang , L. Ma , L. Wang , P. Zhao , C. Wang , J. Ma , Z. Jin , Nano Energy 2020, 73, 104796.

[57] C. Kim , T. Eom , M. S. Jee , H. Jung , H. Kim , B. K. Min , Y. J. Hwang , ACS Catal. 2016, 7, 779.

[58] M. Ma , B. J. Trześniewski , J. Xie , W. A. Smith , Angew. Chem., Int. Ed. 2016, 55, 9748. 10.1002/anie.201604654 27377237

[59] M. S. Jee , H. S. Jeon , C. Kim , H. Lee , J. H. Koh , J. Cho , B. K. Min , Y. J. Hwang , Appl. Catal., B 2016, 180, 372.

[60] Y. W. Choi , F. Scholten , I. Sinev , B. R. Cuenya , J. Am. Chem. Soc. 2019, 141, 5261. 30827111 10.1021/jacs.8b12766 PMC6449802

[61] S. Lamaison , D. Wakerley , J. Blanchard , D. Montero , G. Rousse , D. Mercier , P. Marcus , D. Taverna , D. Giaume , V. Mougel , M. Fontecave , Joule 2020, 4, 395.

[62] W. Zhu , S. Kattel , F. Jiao , J. G. Chen , Adv. Energy Mater. 2019, 9, 1802840.

[63] W. Zhu , L. Zhang , P. Yang , C. Hu , Z. Luo , X. Chang , Z. J. Zhao , J. Gong , Angew. Chem., Int. Ed. 2018, 57, 11544. 10.1002/anie.201806432 29947046

[64] H. Dong , L. Zhang , P. Yang , X. Chang , W. Zhu , X. Ren , Z.‐J. Zhao , J. Gong , Chem. Eng. Sci. 2019, 194, 29.

[65] Z. Han , C. Choi , H. Tao , Q. Fan , Y. Gao , S. Liu , A. W. Robertson , S. Hong , Y. Jung , Z. Sun , Catal. Sci. Technol. 2018, 8, 3894.

[66] J. Pan , Y. Sun , P. Deng , F. Yang , S. Chen , Q. Zhou , H. S. Park , H. Liu , B. Y.u Xia , Appl. Catal., B 2019, 255, 117736.

[67] J. Jiao , R. Lin , S. Liu , W.‐C. Cheong , C. Zhang , Z. Chen , Y. Pan , J. Tang , K. Wu , S.‐F. Hung , H. M. Chen , L. Zheng , Q. Lu , X. Yang , B. Xu , H. Xiao , J. Li , D. Wang , Q. Peng , C. Chen , Y. Li , Nat. Chem. 2019, 11, 222. 30664719 10.1038/s41557-018-0201-x

[68] a) D. R. Kauffman , D. R. Alfonso , D. N. Tafen , C. Wang , Y. Zhou , Y. Yu , J. W. Lekse , X. Deng , V. Espinoza , J. Trindell , O. K. Ranasingha , A. Roy , J. S. Lee , H. L. Xin , J. Phys. Chem. C 2018, 122, 27991;

[69] Y. Mun , S. Lee , A. Cho , S. Kim , J. W. Han , J. Lee , Appl. Catal., B 2019, 246, 82.

[70] H. S. Jeon , J. Timoshenko , F. Scholten , I. Sinev , A. Herzog , F. T. Haase , B. R. Cuenya , J. Am. Chem. Soc. 2019, 141, 19879. 31762283 10.1021/jacs.9b10709 PMC6923792

[71] C. Wang , M. Cao , X. Jiang , M. Wang , Y. Shen , Electrochim. Acta 2018, 271, 544.

[72] M. Feng , X. Wu , H. Cheng , Z. Fan , X. Li , F. Cui , S. Fan , Y. Dai , G. Lei , G. He , J. Mater. Chem. A 2021, 9, 23817.

[73] M. Wang , X. Ren , G. Yuan , X. Niu , Q. Xu , W. Gao , S. Zhu , Q. Wang , J. CO2 Util. 2020, 37, 204.

[74] S. Huo , Z. Weng , Z. Wu , Y. Zhong , Y. Wu , J. Fang , H. Wang , ACS Appl. Mater. Interfaces 2017, 9, 28519. 28786653 10.1021/acsami.7b07707

[75] D. H. Won , H. Shin , J. Koh , J. Chung , H. S. Lee , H. Kim , S. I. Woo , Angew. Chem., Int. Ed. 2016, 55, 9297. 10.1002/anie.201602888 27352078

[76] B. Qin , Q. Zhang , Y. H. Li , G. Yang , F. Peng , ACS Appl. Mater. Interfaces 2020, 12, 30466. 32530600 10.1021/acsami.0c08066

[77] D. L. T. Nguyen , M. S. Jee , D. H. Won , H. Jung , H. S. Oh , B. K. Min , Y. J. Hwang , ACS Sustainable Chem. Eng. 2017, 5, 11377.

[78] K. Liu , J. Wang , M. Shi , J. Yan , Q. Jiang , Adv. Energy Mater. 2019, 9, 1900276.

[79] G. L. Chai , Z. X. Guo , Chem. Sci. 2016, 7, 1268. 29910883 10.1039/c5sc03695j PMC5975832

[80] Y. Guo , H. Yang , X. Zhou , K. Liu , C. Zhang , Z. Zhou , C. Wang , W. Lin , J. Mater. Chem. A 2017, 5, 24867.

[81] W. Kou , Y. Zhang , J. Dong , C. Mu , L. Xu , ACS Appl. Energy Mater. 2020, 3, 1875.

[82] A. S. Varela , W. Ju , A. Bagger , P. Franco , J. Rossmeisl , P. Strasser , ACS Catal. 2019, 9, 7270.

[83] C. Zhang , S. Yang , J. Wu , M. Liu , S. Yazdi , M. Ren , J. Sha , J. Zhong , K. Nie , A. S. Jalilov , Z. Li , H. Li , B. I. Yakobson , Q. Wu , E. Ringe , H. Xu , P. M. Ajayan , J. M. Tour , Adv. Energy Mater. 2018, 8, 1703487.

[84] H. Zhang , J. Li , S. Xi , Y. Du , X. Hai , J. Wang , H. Xu , G. Wu , J. Zhang , J. Lu , J. Wang , Angew. Chem., Int. Ed. 2019, 58, 14871. 10.1002/anie.201906079 31368619

[85] F. Pan , B. Li , E. Sarnello , S. Hwang , Y. Gang , X. Feng , X. Xiang , N. M. Adli , T. Li , D. Su , G. Wu , G. Wang , Y. Li , Nano Energy 2020, 68, 104384.

[86] Y. Pan , R. Lin , Y. Chen , S. Liu , W. Zhu , X. Cao , W. Chen , K. Wu , W.‐C. Cheong , Y. Wang , L. Zheng , J. Luo , Y. Lin , Y. Liu , C. Liu , J. Li , Q. Lu , X. Chen , D. Wang , Q. Peng , C. Chen , Y. Li , J. Am. Chem. Soc. 2018, 140, 4218. 29517907 10.1021/jacs.8b00814

[87] J. Shen , M. J. Kolb , A. J. Göttle , M. T. M. Koper , J. Phys. Chem. C 2016, 120, 15714.

[88] H. J. Zhu , M. Lu , Y. R. Wang , S. J. Yao , M. Zhang , Y.‐H. Kan , J. Liu , Y. Chen , S. L. Li , Y. Q. Lan , Nat. Commun. 2020, 11, 497. 31980641 10.1038/s41467-019-14237-4 PMC6981265

[89] G. Zhu , Y. Li , H. Zhu , H. Su , S. H. Chan , Q. Sun , ACS Catal. 2016, 6, 6294.

[90] X. M. Hu , M. H. Rønne , S. u. Pedersen , T. Skrydstrup , K. Daasbjerg , Angew. Chem., Int. Ed. 2017, 56, 6468. 10.1002/anie.201701104 28466962

[91] M. Zhu , J. Chen , L. Huang , R. Ye , J. Xu , Y. F. Han , Angew. Chem., Int. Ed. 2019, 58, 6595. 10.1002/anie.201900499 30689279

[92] X. Zhang , Y. Wang , M. Gu , M. Wang , Z. Zhang , W. Pan , Z. Jiang , H. Zheng , M. Lucero , H. Wang , G. E. Sterbinsky , Q. Ma , Y. G. Wang , Z. Feng , J. Li , H. Dai , Y. Liang , Nat. Energy 2020, 5, 684.

[93] X. Yang , J. Cheng , X. Xuan , N. Liu , J. Liu , ACS Sustainable Chem. Eng. 2020, 8, 10536.

[94] Z. Guo , N. Xiao , H. Li , Y. Wang , C. Li , C. Liu , J. Xiao , J. Bai , S. Zhao , J. Qiu , J. CO2 Util. 2020, 38, 212.

[95] Y. Li , S. L. Zhang , W. Cheng , Y. Chen , D. Luan , S. Gao , X. W. D. Lou , Adv. Mater. 2021, 33, 2105204.

[96] C. Zhao , Y. Wang , Z. Li , W. Chen , Q. Xu , D. He , D. Xi , Q. Zhang , T. Yuan , Y. Qu , J. Yang , F. Zhou , Z. Yang , X. Wang , J. Wang , J. Luo , Y. Li , H. Duan , Y. Wu , Y. Li , Joule 2019, 3, 584.

[97] N. T. Suen , Z. R. Kong , C. S. Hsu , H. C. Chen , C. W. Tung , Y. R. Lu , C. L. Dong , C. C. Shen , J. C. Chung , H. M. Chen , ACS Catal. 2019, 9, 5217.

[98] Y. Wang , H. Shen , K. J. T. Livi , D. Raciti , H. Zong , J. Gregg , M. Onadeko , Y. Wan , A. Watson , C. Wang , Nano Lett. 2019, 19, 8461. 31671267 10.1021/acs.nanolett.9b02748

[99] Q. Yang , X. Liu , W. Peng , Y. Zhao , Z. Liu , M. Peng , Y. R. Lu , T. S. Chan , X. Xu , Y. Tan , J. Mater. Chem. A 2021, 9, 3044.

[100] Y. Chen , Z. Fan , J. Wang , C. Ling , W. Niu , Z. Huang , G. Liu , B. Chen , Z. Lai , X. Liu , B. Li , Y. Zong , L. Gu , J. Wang , X. Wang , H. Zhang , J. Am. Chem. Soc. 2020, 142, 12760. 32551635 10.1021/jacs.0c04981

[101] B. Zhang , J. Zhang , M. Hua , Q. Wan , Z. Su , X. Tan , L. Liu , F. Zhang , G. Chen , D. Tan , X. Cheng , B. Han , L. Zheng , G. Mo , J. Am. Chem. Soc. 2020, 142, 13606. 32658474 10.1021/jacs.0c06420

[102] C. Tang , J. Shi , X. Bai , A. Hu , N. Xuan , Y. Yue , T. Ye , B. Liu , P. Li , P. Zhuang , J. Shen , Y. Liu , Z. Sun , ACS Catal. 2020, 10, 2026.

[103] Y. Gao , Q. Wu , X. Liang , Z. Wang , Z. Zheng , P. Wang , Y. Liu , Y. Dai , M. H. Whangbo , B. Huang , Adv. Sci. 2020, 7, 1902820. 10.1002/advs.201902820 PMC7080533 32195095

[104] Z. Yin , C. Yu , Z. Zhao , X. Guo , M. Shen , N. Li , M. Muzzio , J. Li , H. Liu , H. Lin , J. Yin , G. Lu , D. Su , S. Sun , Nano Lett. 2019, 19, 8658. 31682758 10.1021/acs.nanolett.9b03324

[105] H. Yang , Y. Wu , G. Li , Q. Lin , Q. Hu , Q. Zhang , J. Liu , C. He , J. Am. Chem. Soc. 2019, 141, 12717. 31348650 10.1021/jacs.9b04907

[106] H. Xu , D. Rebollar , H. He , L. Chong , Y. Liu , C. Liu , C. J. Sun , T. Li , J. V. Muntean , R. E. Winans , D. J. Liu , T. Xu , Nat. Energy 2020, 5, 623.

[107] C. J. Chang , S. C. Lin , H. C. Chen , J. Wang , K. J. Zheng , Y. Zhu , H. M. Chen , J. Am. Chem. Soc. 2020, 142, 12119. 32558560 10.1021/jacs.0c01859

[108] J. Huang , M. Mensi , E. Oveisi , V. Mantella , R. Buonsanti , J. Am. Chem. Soc. 2019, 141, 2490. 30657662 10.1021/jacs.8b12381

[109] L. R. L. Ting , O. Piqué , S. Y. Lim , M. Tanhaei , F. Calle‐Vallejo , B. S. Yeo , ACS Catal. 2020, 10, 4059.

[110] J. Fu , W. Zhu , Y. Chen , Z. Yin , Y. Li , J. Liu , H. Zhang , J. J. Zhu , S. Sun , Angew. Chem., Int. Ed. 2019, 58, 14100. 10.1002/anie.201905318 31314934

[111] L. Xiong , X. Zhang , H. Yuan , J. Wang , X. Yuan , Y. Lian , H. Jin , H. Sun , Z. Deng , D. Wang , J. Hu , H. Hu , J. Choi , J. Li , Y. Chen , J. Zhong , J. Guo , M. H. Rümmerli , L. Xu , Y. Peng , Angew. Chem., Int. Ed. 2021, 60, 2508. 10.1002/anie.202012631 33009695

[112] Z. Wang , Q. Yuan , J. Shan , Z. Jiang , P. Xu , Y. Hu , J. Zhou , L. Wu , Z. Niu , J. Sun , T. Cheng , W. A. Goddard , J. Phys. Chem. Lett. 2020, 11, 7261. 32700911 10.1021/acs.jpclett.0c01261

[113] M. S. Xie , B. Y. Xia , Y. Li , Y. Yan , Y. Yang , Q. Sun , S. H. Chan , A. Fisher , X. Wang , Energy Environ. Sci. 2016, 9, 1687.

[114] Y. Qiu , H. Zhong , W. Xu , T. Zhang , X. Li , H. Zhang , J. Mater. Chem. A 2019, 7, 5453.

[115] Z. Han , R. Kortlever , H. Y. Chen , J. C. Peters , T. Agapie , ACS Cent. Sci. 2017, 3, 853. 28852699 10.1021/acscentsci.7b00180 PMC5571460

[116] A. Thevenon , A. Rosas‐Hernández , J. C. Peters , T. Agapie , Angew. Chem., Int. Ed. 2019, 58, 16952. 10.1002/anie.201907935 31538402

[117] Y. Song , R. Peng , D. K. Hensley , P. V. Bonnesen , L. Liang , Z. Wu , H. M. Meyer , M. Chi , C. Ma , B. G. Sumpter , A. J. Rondinone , ChemistrySelect 2016, 1, 6055.

[118] Y. S. Cheng , X. P. Chu , M. Ling , N. Li , K. L. Wu , F. H. Wu , H. Li , G. Yuan , X. W. Wei , Catal. Sci. Technol. 2019, 9, 5668.

[119] G. Shi , L. Yu , X. Ba , X. Zhang , J. Zhou , Y. Yu , Dalton Trans. 2017, 46, 10569. 28106214 10.1039/c6dt04381j

[120] Y. Wang , Z. Chen , P. Han , Y. Du , Z. Gu , X. Xu , G. Zheng , ACS Catal. 2018, 8, 7113.

[121] S. Back , Y. Jung , ACS Energy Lett. 2017, 2, 969.

[122] S. Payra , S. Shenoy , C. Chakraborty , K. Tarafder , S. Roy , ACS Appl. Mater. Interfaces 2020, 12, 19402. 32270996 10.1021/acsami.0c00521

[123] A. R. Paris , A. B. Bocarsly , ACS Catal. 2017, 7, 6815.

[124] S. K. Kim , Y.‐J. Zhang , H. Bergstrom , R. Michalsky , A. Peterson , ACS Catal. 2016, 6, 2003.

[125] X. Liu , H. Yang , J. He , H. Liu , L. Song , L. Li , J. Luo , Small 2018, 14, 1704049. 10.1002/smll.201704049 29517837

[126] X. Sun , X. Kang , Q. Zhu , J. Ma , G. Yang , Z. Liu , B. Han , Chem. Sci. 2016, 7, 2883. 30090281 10.1039/c5sc04158a PMC6054036

[127] S. Tang , X. Zhou , S. Zhang , X. Li , T. Yang , W. Hu , J. Jiang , Y. Luo , ACS Appl. Mater. Interfaces 2018, 11, 906. 30525373 10.1021/acsami.8b18505

[128] D. Pan , X. Ye , Y. Cao , S. Zhu , X. Chen , M. Chen , D. Zhang , G. Li , Appl. Surf. Sci. 2020, 511, 145497.

[129] Y. Li , H. Yang , X. Hu , H. Tian , M. Gao , D. Zhang , Z. Li , D. Yang , ChemElectroChem 2019, 6, 3782.

[130] B. Paul , N. Manwar , P. Bhanja , S. Sellaiyan , S. K. Sharma , R. Khatun , S. Jain , R. Bal , J. CO2 Util. 2020, 41, 101284.

[131] J. Liu , H. Shi , Q. Shen , C. Guo , G. Zhao , Green Chem. 2017, 19, 5900.

[132] Z. Li , H. Cheng , Y. Li , W. Zhang , Y. Yu , ACS Sustainable Chem. Eng. 2019, 7, 4325.

[133] L. Wei , J. Lin , S. Xie , W. Ma , Q. Zhang , Z. Shen , Y. Wang , Nanoscale 2019, 11, 12530. 31179477 10.1039/c9nr02786f

[134] L. Chen , Z. Wang , P. Kang , Chin. J. Catal. 2018, 39, 413.

[135] W. Lu , F. Ju , K. Yao , X. Wei , Ind. Eng. Chem. Res. 2020, 59, 4348.

[136] A. Navaee , A. Salimi , J. Colloid Interface Sci. 2017, 505, 241. 28578287 10.1016/j.jcis.2017.05.103

[137] L. Zhang , H. Cao , Y. Lu , H. Zhang , G. Hou , Y. Tang , G. Zheng , J. Colloid Interface Sci. 2020, 568, 198. 32088450 10.1016/j.jcis.2020.01.082

[138] Y. Ru , L. Yang , Y. Li , W. Jiang , Y. Li , Y. Luo , L. Yang , T. Li , S. Luo , J. Mater. Sci. 2018, 53, 10351.

[139] J. Liu , H. Shi , Q. Shen , C. Guo , G. Zhao , Appl. Catal., B 2017, 210, 368.

[140] J. Wang , Y. Guan , X. Yu , Y. Cao , J. Chen , Y. Wang , B. Hu , H. Jing , iScience 2020, 23, 100768. 31887657 10.1016/j.isci.2019.100768 PMC6941872

[141] M. Zhang , J. Cheng , X. Xuan , J. Zhou , K. Cen , Chem. Eng. J. 2017, 322, 22.

[142] Y. Xu , S. Wang , J. Yang , B. Han , R. Nie , J. Wang , J. Wang , H. Jing , Nano Energy 2018, 51, 442.

[143] J. Wang , J. Ma , Q. Zhang , Y. Chen , L. Hong , B. Wang , J. Chen , H. Jing , Appl. Catal., B 2021, 285, 119781.

[144] L. Fan , C. Xia , F. Q. Yang , J. Wang , H. T. Wang , Y. Y. Lu , Sci. Adv. 2020, 6, eaay3111. 32128404 10.1126/sciadv.aay3111 PMC7034982

[145] T. Burdyny , W. A. Smith , Energy Environ. Sci. 2019, 12, 1442.

[146] T. Möller , W. Ju , A. Bagger , X. Wang , F. Luo , T. N. Thanh , A. S. Varela , J. Rossmeisl , P. Strasser , Energy Environ. Sci. 2019, 12, 640.

[147] J. J. Lv , M. Jouny , W. Luc , W. Zhu , J. J. Zhu , F. Jiao , Adv. Mater. 2018, 30, 1803111. 10.1002/adma.201803111 30368917

[148] W. W. Lu , Y. Zhang , J. J. Zhang , P. Xu , Ind. Eng. Chem. Res. 2020, 59, 5536.

